# Recent advances microRNAs and metabolic reprogramming in colorectal cancer research

**DOI:** 10.3389/fonc.2023.1165862

**Published:** 2023-07-27

**Authors:** Bin Xiong, Qiaoyi Huang, Huida Zheng, Shu Lin, Jianhua Xu

**Affiliations:** ^1^ Department of Gastrointestinal Surgery, The Second Affiliated Hospital of Fujian Medical University, Quanzhou, Fujian, China; ^2^ Department of Gynaecology and Obstetrics, The Second Affiliated Hospital of Fujian Medical University, Quanzhou, Fujian, China; ^3^ Centre of Neurological and Metabolic Research, The Second Affiliated Hospital of Fujian Medical University, Quanzhou, Fujian, China; ^4^ Group of Neuroendocrinology, Garvan Institute of Medical Research, Sydney, NSW, Australia

**Keywords:** colorectal cancer, miRNA, metabolism reprogramming, signaling/signaling pathways, chemotherapy resistance

## Abstract

Colorectal cancer (CRC) is a cancer with the highest incidence and mortality. Alteration of gene expression is the main pathophysiological mechanism of CRC, which results in disturbed signaling pathways and cellular metabolic processes. MicroRNAs are involved in almost all pathophysiological processes and are correlative with colorectal cancer metabolism, proliferation, and chemotherapy resistance. Metabolic reprogramming, an important feature of cancer, is strongly correlative with the development and prognosis of cancers, including colorectal cancer. MicroRNAs can target enzymes involved in metabolic processes, thus playing a regulatory role in tumor metabolism. The disorder of the signaling pathway is another characteristic of tumor, which induces the occurrence and proliferation of tumors, and is closely correlative with the prognosis and chemotherapy resistance of tumor patients. MicroRNAs can target the components of the signaling pathways to regulate their transduction. Understanding the function of microRNAs in the occurrence and proliferation of CRC provides novel insights into the optimal treatment strategies, prognosis, and development of diagnosis in CRC. This article reviews the relationship between CRC and microRNA expression and hopes to provide new options for the diagnosis and treatment of CRC.

## 1 Introduction

Colorectal cancer (CRC) is one of the most common malignancies globally (1). In some developed countries, its morbidity and mortality have declined with the availability of screening tests (fecal occult blood test and colonoscopy). Nonetheless, its incidence and mortality continues to increase worldwide (2). Notably, the morbidity of CRC increases with age, and the rates of early-onset colorectal cancer are increasing (3).

Environmental and genetic factors are two of its important causes. Most CRC cases are sporadic and can arise from lifestyle factors, including physical inactivity, high fat diet, smoking, and obesity (4). Genetic susceptibility is a fundamental cause of CRC; approximately 30% of cases are related to genetic factors, some of which are gene mutations in signaling pathways (5). Dysregulation of signaling pathways directly or indirectly promotes metabolic reprogramming in CRC.

Metabolism reprogramming, including aerobic glycolysis, disturbances in lipid synthesis and decomposition, and enhancement of amino acid metabolism, is a crucial characteristic of CRC. Abnormal metabolism meets the energy and nutritional needs of CRC cells and promotes their proliferation (6). Hyperactivated energy metabolism and dysregulated signaling pathways lead to poor prognosis of CRC.

CRC generally develops slowly: the period from tumor occurrence to the manifestation of clinical symptoms is long. CRC patients may initially have no significant symptoms. When they visit the hospital with complaints of abdominal pain, vomiting, and bowel obstruction, patients are often in the middle or advanced CRC stage, and a poor prognosis is often expected (7).

Therefore, early screening of CRC is essential. Colonoscopy is the best strategy for CRC diagnosis, but its high cost and invasiveness have hindered its widespread use (1). Therefore, a new, low-cost, non-invasive screening test is required. Surgery is the most effective treatment for CRC, mainly involving minimally invasive laparoscopic surgery (8). Preoperative neoadjuvant therapy to reduce tumor size for surgical resection significantly prolongs disease-free survival (DFS) after surgery (9). In addition, immunotherapy, chemotherapy, radiotherapy, and targeted therapy are essential methods of treatment for CRC.

MicroRNAs (miRNAs) were first discovered in the 1990s in *Caenorhabditis elegans* (10). They are a type of single-stranded, non-coding RNA that comprise only 1% of the human genome but are involved in almost all pathophysiological changes, including metabolic reprogramming and signaling pathway disorders in cancer (11). More than 30% of protein-coding genes in the human genome are regulated by miRNAs (12). MiRNAs are involved in cell growth, proliferation, differentiation, and apoptosis, and regulate tumor progression. Furthermore, miRNAs can silence messenger RNA (mRNA) and regulate genes by binding to the 3′ untranslated regions (3′ UTR) of mRNA. MiRNAs can regulate metabolic reprogramming and tumor-related signaling pathways to mediate tumor progression and metastasis, and some miRNAs may also influence drug resistance in tumors (13). Based on these characteristics, researchers are exploring the efficacy of miRNAs as biomarkers for the diagnosis, treatment, and prognosis of CRC.

Here, we review the role of miRNAs in the alteration of metabolic reprogramming and transduction of signaling pathways and how these processes interfere with the occurrence and proliferation of CRC. This review may help researchers improve the screening methods for and prognosis of CRC.

### 1.1 MicroRNAs and metabolic reprogramming

**Figure 1:**
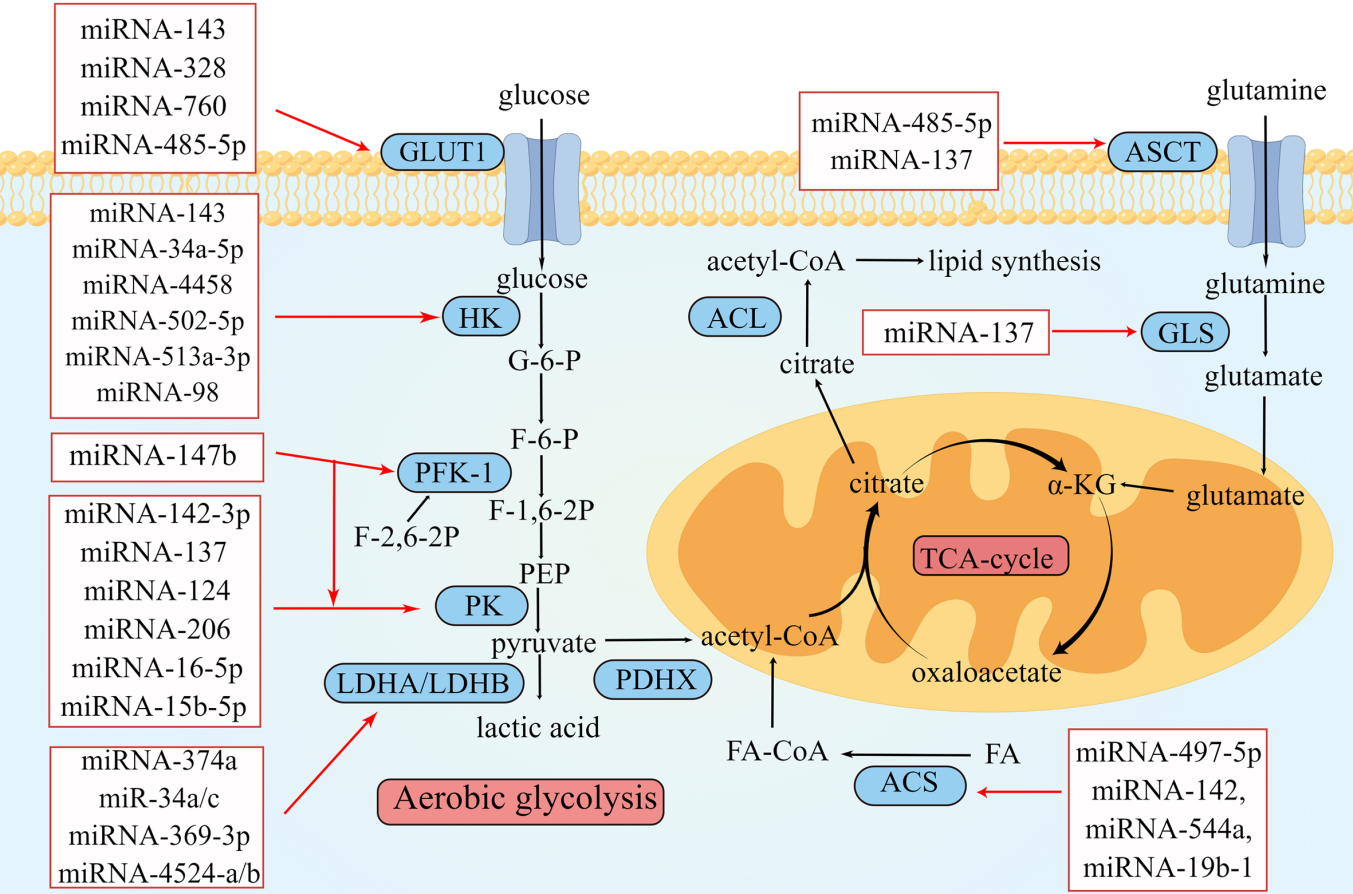
Overview of miRNAs in metabolism reprogramming of CRC. Schematic illustration shows microRNAs that regulate metabolic reprogramming in colorectal cancer by targeting three metabolically related enzymes. The black arrows depict the transformation of substances during metabolism, and the red arrows depict the inhibitory effect of miRNAs on enzymes involved in metabolism. The expression of miRNAs in red squares decreased in colorectal cancer. The illustration was generated by Figdraw.

Metabolism reprogramming is a critical hallmark of CRC. It refers to the alteration of metabolism that occurs as CRC cells adapt to the tumor microenvironment (TME). These changes satisfy the energy and nutrient demands of tumor cells and sustain the rapid proliferation rate of CRC (6). MiRNAs are important regulators of metabolic reprogramming. They regulate metabolic processes and reshape TME by promoting or inhibiting biomolecules (enzymes or transporters) involved in metabolic processes. The crosstalk between tumor metabolites and TME components is tightly related to proliferation, metastasis, and drug resistance in CRC. In this review, we mainly focus on recent studies of miRNA-induced metabolic reprogramming in glucose, lipid, and amino acid metabolism (**Table 1** and **Figure 1**).

**Table 1:** List of microRNAs involved in metabolism reprogramming.

Micro RNA	Expression in CRC	Target	Description	Effect on CRC	Ref
miRNA-143	↓	GLUT1	Down-regulate the expression of GLUT1 and inhibit aerobic glycolysis	Inhibit proliferation	(14)
		HK2	Down-regulate the expression of HK2 and inhibit aerobic glycolysis	Inhibit proliferation	(15)
miRNA-328	↓	SLC2A1 /GLUT1	Down-regulate the expression of GLUT1 and inhibit aerobic glycolysis	Inhibit proliferation	(16)
miRNA-760	↓	GLUT1	Down-regulate the expression of GLUT1 and inhibit aerobic glycolysis	Inhibit proliferation	(17)
miRNA-34a-5p	↓	HK1	lncARSR sponges miRNA-34a-5p to promote the expression of HK-1	Inhibit migration and invasion	(18)
miRNA-4458	↓	HK2	inhibit glycolysis and lactate production via directly targeting HK2 mRNA	inhibit proliferation	(19)
miRNA-502-5p	↓	MYO6 /HK2	Target MYO6 and down-regulate the expression of HK2, inhibit aerobic glycolysis	Inhibit proliferation, migration and invasion	(20)
miRNA-513a-3p	↓	HK2	Down-regulate the expression of HK2 and inhibit aerobic glycolysis	Inhibit proliferation	(21)
miRNA-98	↓	HK2	Down-regulate the expression of HK2 and inhibit aerobic glycolysis	Inhibit proliferation	(22)
miRNA-147b	↓	PKM2 /PFK1	Down-regulate the expression of PKM2, PFK1 and inhibit aerobic glycolysis	Inhibit proliferation, migration and invasion	(23)
miRNA-488	↓	PFKF3B3	Target PFKFB3 and lessen its mRNA level, inhibit aerobic glycolysis	Inhibit proliferation, migration, invasion and drug resistance	(24)
miRNA-142-3p	↓	PKM2	Down-regulate the expression of PKM2 via binding to the 3′-UTR of PKM2 mRNA	Inhibit proliferation, migration and invasion	(25)
miRNA-137	↓	PKM2/PKM1	Down-regulate the expression of PKM2, PFK1 and inhibit aerobic glycolysis	Inhibit proliferation, migration, invasion and drug resistance	(26)
		ASCT2	Down-regulate the expression of ASCT2 to reduce the intake of glutamine	Inhibit proliferation	(27)
		GLS1	Down-regulate the expression of GLS1 to inhibit the decomposition and transformation of glutamine	Inhibit proliferation	(28)
miRNA-124	↓	PTB1 /PKM1 /PKM2	Target PTB1 to increase the PKM1/PKM2 ratio, and inhibit aerobic glycolysis	Inhibit proliferation	(29)
miRNA-206	↓	hnRNPA1 /PKM2	Target hnRNPA1 to increase the PKM1/PKM2 ratio, and inhibit aerobic glycolysis	Inhibit proliferation	(30)
miRNA-16-5p/ miRNA-15b-5p	↓	ALDH1A3 /PKM2	Target ALDH1A3 to down-regulate the expression of PKM2, and inhibit aerobic glycolysis	Inhibit proliferation	(31)
miRNA-374a, miRNA-34a, miR-34c, miRNA-369-3p, miRNA-4524-a/b	↓	LDHA	suppress aerobic glycolysis through inhibition of LDHA	Inhibit proliferation	(32)
miRNA-1224-5p	↓	FOXM1	Target FOXM1 and inhibit CRC progression and cell glycolysis	Inhibit proliferation	(33)
miRNA-874-3p	↓	FOXM1	Target the 3′UTR of FOXM1, and inhibit the expression of lncRNA MCF2L-AS1(deficiency of MCF2L-AS1 led to substantial reduction in the protein levels of GLUT1 and LDHA)	Inhibit proliferation, migration and invasion	(34)
miRNA-485-5p	↓	CKS1B /GLUT1 /LDHA	Target the 3′UTR of CKS1B, and down-regulate the expression of GLUT1 and LDHA	Inhibit proliferation, migration and invasion	(35)
		SLC38A1	Down-regulate the expression of SLC38A1 and inhibit the transport of amino acid	inhibit proliferation and migration	(36)
miRNA-181a	↑	PTEN/AKT pathway	inhibit the expression of PTEN, lead to an increase of phosphorylated AKT, promote aerobic glycolysis	Promote proliferation	(37)
miRNA-181d	↑	CRY2 /FBXL3	inhibit the expression of CRY2 and FBXL3, promote aerobic glycolysis	promote proliferation, migration and invasion	(38)
miRNA-26a	↑	PDHX	Down-regulate the expression of PDHX and promote aerobic glycolysis	Promote proliferation	(39)
miRNA-142-5p	↑	SDHB	Down-regulate the expression of SDHB, increase glucose consumption and lactate production	promote proliferation	(40)
miRNA-497-5p	↓	ACSL5	Down-regulate ACSL5 and suppress fatty acyl-CoA production	Inhibit proliferation, migration and invasion	(41)
miRNA-142, miRNA-544a, miRNA-19b-1	↓	ACSL1 /ACSL4 /SCD	Target the 3′UTR of ACSL1, ACSL4, and SCD, then regulate lipid metabolism	Inhibit proliferation and invasion	(42)
miRNA-146-5p	↑	HOXC10	affect WAT browning and cause cachexia by targeting HOXC 10	prompt adipose tissue browning and accelerate lipolysis	(43)

### 1.2 Glucose metabolism

**Figure 2:**
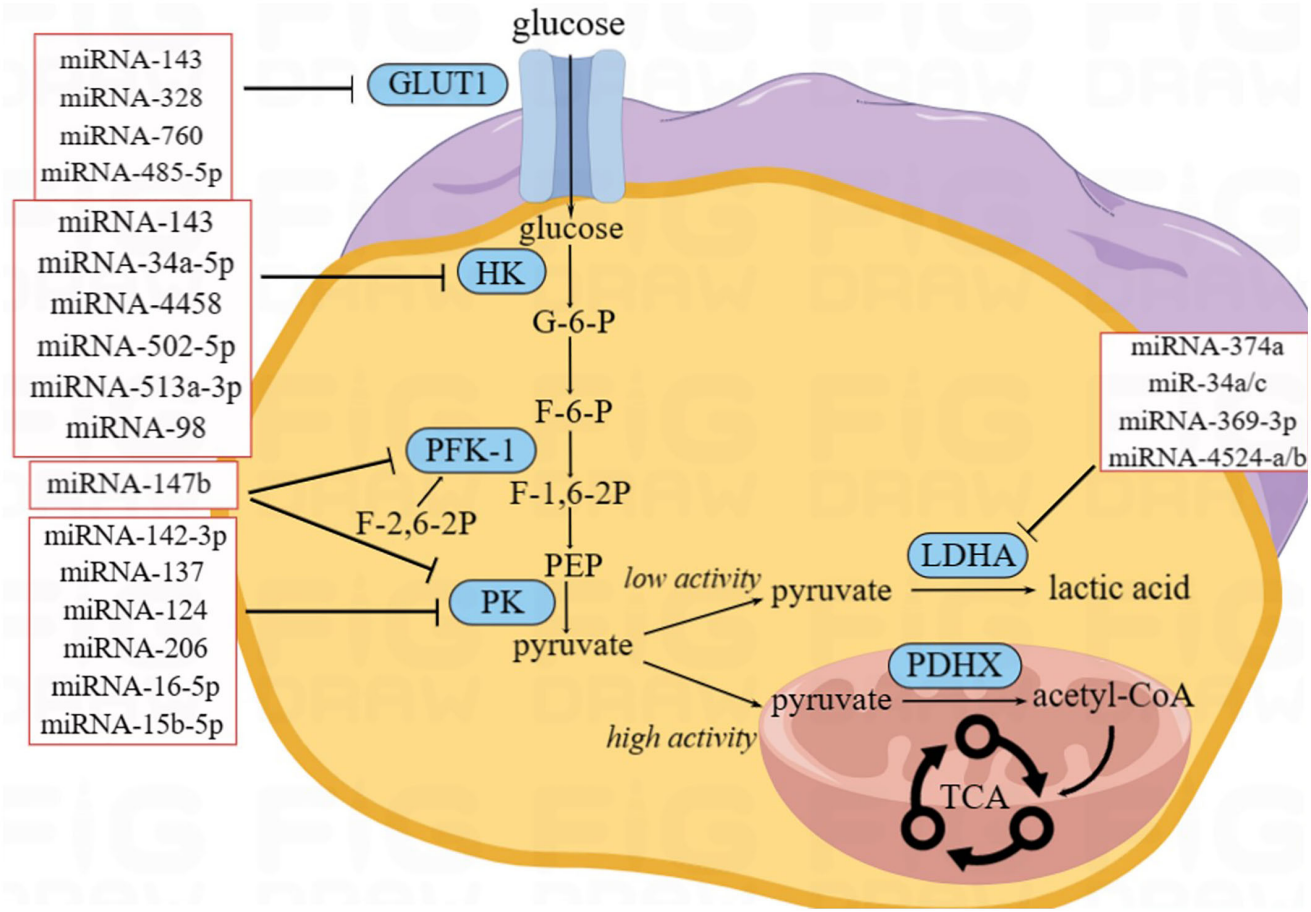
Effect of miRNAs on glycolysis.

Glucose is a vital energy source for the human body. Therefore, to meet the energy needs of tumor growth, abnormal glucose metabolism often occurs in CRC cells. Unlike most normal tissues, tumor cells can obtain ATP through glycolysis, even in the presence of oxygen (44). This phenomenon is termed aerobic glycolysis, which is also known as the Warburg effect (**Figure 2**).

Whereas oxidative phosphorylation of a glucose molecule in normal tissues produces 36 ATP molecules, aerobic glycolysis produces only two (44). However, aerobic glycolysis in tumor cells may occur at much higher rates than oxidative phosphorylation (45). Therefore, it can meet the enormous energy demands of tumor proliferation.

The first step in glucose metabolism is transporting glucose from the extracellular to the intracellular space through glucose transporter 1 (GLUT1) (46). GLUT1 is upregulated in tumor tissues to meet the demand for the large amount of glucose required for aerobic glycolysis (46). During this process, some miRNAs can directly or indirectly act on GLUT1. One study showed that the expression level of miRNA-143 in CRC was significantly related to tumor size and that its overexpression 143 could inhibit the function of GLUT1, thereby inhibiting the proliferation of CRC cells (14). Santasusagna et al. (16) demonstrated that miRNA-328 could inhibit the solute carrier family 2 member 1 (SLC2A1), which encodes the GLUT1 protein, thus downregulating the expression of GLUT1 in tumors and inhibiting tumor proliferation. Increased circRNA DENND4C expression in CRC downregulates the expression of its target miRNA-760, leading to the overexpression of GLUT1, which would accelerate glycolysis and ultimately promote the proliferation of CRC (17). The expression of several of the abovementioned miRNAs was negatively correlated with that of GLUT1; its downregulation could promote CRC proliferation.

The second step in glucose metabolism is hexose phosphorylation, where glucose is converted to glucose-6-phosphate (G-6-P) by hexokinase (HK). Four isoenzymes of hexokinase, HK-1 to HK-4, have been found in mammals, of which HK-1 and HK-2 are found on the outer mitochondrial membrane. In various cancers, including CRC, HK-1 and HK-2 are upregulated to meet the high metabolic state of tumor cells and accelerate tumor cell proliferation, migration, and invasion (47). Many miRNAs target HK1 or HK2 to affect glucose metabolism in CRC and interfere with tumor proliferation. Li et al. (18) found that lncARSR sponged miRNA-34a-5p to promote the expression of HK-1. MiRNA-4458 targets and inhibits HK2 to suppress glycolysis and lactate production (19). I_circ_0000231 functioned as a sponge of miR-502-5p and promoted HK2 protein expression; this indicated that miRNA-502-5p repressed HK2 by inhibiting the expression IHsa_circ_0000231 and ultimately inhibited aerobic glycolysis (20). MiRNA-143, miRNA-513a-3p, and miRNA-98 have low expression in CRC; these miRNAs could target HK2 and downregulate its expression, thereby inhibiting aerobic glycolysis in CRC (15, 21, 22).

G-6-P is catalyzed by hexose phosphate isomerase to form fructose-6-phosphate (F-6-P), which becomes fructose-1,6-bisphosphate (F-1,6-P_2_) via phosphofructokinase 1 (PFK1). PFK1 is the main rate-limiting enzyme in glycolysis and is involved in the second phosphorylation reaction during glycolysis (48). F-2,6-2P, an isomer of F-1,6-2P, activates PFK1 and accelerates the production of F-1,6-2P (48). The intracellular concentration of F-2,6-2P is regulated by 6-phosphofructo-2-kinase/fructose-2,6-biphosphatase 3 (PFKFB3) (49). Cui et al. (23) found that miRNA-147b was downregulated in CRC, resulting in the upregulation of PFK1 and PKM2. Deng et al. (24) reported that miRNA-488 targeted PFKFB3 in CRC and that miRNA-488 and PFKFB3 inhibited each other. They suggested that miRNA-488 negatively regulates glycolysis in CRC, which may be related to the regulation of F-2,6-2P by miRNA-488 and PFKFB3.

The third step in glycolysis is the production of pyruvate. One molecule of F-1,6-2P generates two molecules of phosphoenolpyruvate (PEP), which then generate two molecules of pyruvate through pyruvate kinase (PK). PK is a key enzyme of glucose metabolism and can be divided into PKM1 and PKM2 (50). PKM2 is expressed in tissues with abnormal differentiation or proliferation. In tumor cells, PKM2 can catalyze the conversion of PEP to pyruvate under aerobic conditions, which plays a vital role in aerobic glycolysis (50). Ren et al. (25) found that miRNA-142-3p is lowly expressed in CRC; however, its overexpression inhibited PKM2 by binding to the 3′ UTR of PKM2 mRNA, thus inhibiting proliferation, migration, and invasion of CRC. Wang et al. (51) reported that the circulaINA hsa_circ_0005963 could enhance the expression of PKM2 by sponging miRNA-122, thereby promoting aerobic glycolysis and chemotherapy resistance. Zheng et al. (26) found that the lncRNA XIST–miRNA-137–PKM axis could control the ratio of PKM1 and PKM2 in CRC. After silencing XIST, the expression of miRNA-137 was upregulated, and the percentage of PKM2/PKM1 was decreased, inhibiting glycolysis.

Taniguchi et al. (29) revealed that miRNA-124 is a PK splicer that induces the transform of PKM2 to PKM1 and inhibits tumor proliferation. Fu et al. (30) found that miRNA-206 could inhibit hnRNPA1 and convert PKM2 to PKM1, thereby inhibiting aerobic glycolysis. Furthermore, CuET, an intermediate metabolized *in vivo* by disulfiram (DSF), inhibited ALDH1A3 by enhancing the expression of miRNA-16-5p and miRNA-15b-5p (31). However, ALDH1A3 could inhibit the ubiquitination of PKM2. Therefore, when miRNA-16-5p and miRNA-15b-5p levels are elevated, the decomposition of PKM2 is no longer restricted, and aerobic glycolysis is inhibited.

In the fourth step of aerobic glycolysis, pyruvate is catalyzed by lactate dehydrogenase (LDH) to produce lactic acid in tumor cells, rather being converted to acetyl-CoA in normal tissues under aerobic conditions. The tetramer proteins of LDHA and LDHB include five LDH isoenzymes (LDH1–5). LDHA is upregulated in several cancers and is close to aerobic glycolysis (52). One study reported that the expression of miRNA-34a, miRNA-34c, miRNA-369-3p, miRNA-374a, and miRNA-4524a/b was negatively correlated with that of LDHA in CRC (32). Only the *LDHA* knockout cannot prevent pyruvate conversion to lactic acid because LDHB can replace the function of LDHA in the absence of LDHA (53). This suggests that simultaneous inhibition of LDHA and LDHB could attenuate tumor aerobic glycolysis. miRNA-335-5p could downregulate the expression of LDHB, thereby inhibiting the proliferation and metastasis of CRC cells (54). After the knockout of both LDHA and LDHB, aerobic glycolysis was disrupted, tumor growth was delayed but not abolished, and glucose metabolism was transformed to oxidative phosphorylation, indicating that tumor metabolism is a complex and flexible process and the Warburg effect is not irreplaceable.

In addition, miRNAs can regulate glucose metabolism in CRC by affecting the expression of certain genes. For example, the forkhead Box M1 (*FOXM1*) is a protein-coding gene that controls cell proliferation. Both miRNA-1224-5p and miRNA-874-3p have low expression in CRC and inhibit CRC progression and cell glycolysis by targeting the 3′ UTR of FOXM1 (33, 34). Wang et al. (35) found that the CDC28 protein kinase regulatory subunit 1B (CKS1B) is a direct target of miRNA-485-5p. The interaction between CKS1B and miRNA-485-5p regulates the expression of GLUT1 and LDHA. MiRNA-485-5p inhibits the development of CRC by reducing the expression of CKS1B.

Some miRNAs are also upregulated in CRC and contributing to promote tumor proliferation, metastasis, and invasion. The levels of miRNA-181a/d are abnormally elevated in CRC, promoting aerobic glycolysis (37, 38). Pyruvate dehydrogenase complex X (*PDHX*) is a protein-coding gene that catalyzes pyruvate conversion to acetyl-CoA. MiRNA-26a suppresses the expression of PDHX by targeting the 3′ UTR mRNA of PDHX and promotes aerobic glycolysis to meet the increased energy demands in CRC (39). Liu et al. (40) found that abnormally upregulated miRNA-142-5p in CRC decreased the expression of SDHB and promoted aerobic glycolysis by increasing glucose consumption and lactate production.

### 1.3 Lipid metabolism

Abnormal lipid metabolism is one of the fundamental metabolic features of cancer. Lipids participate in regulating tumor proliferation and metastasis. β-oxidation is one of the critical steps in lipid metabolism, which produces acetyl-CoA and provides energy for tumor growth and proliferation (55). Some miRNAs can regulate tumor cell proliferation and progression by influencing the enzymes involved in β-oxidation. β-oxidation first requires the activation of fatty acids, which generates fatty acid CoA (FA-CoA) under the catalysis of acyl-CoA synthase (ACS). ACSL5, a subtype of ACS, is upregulated in CRC and related to the poor prognosis of CRC (41). One study showed that miRNA-497-5p negatively regulated ACSL5 (41). They proposed that miRNA-497-5p may act as a therapeutic strategy for regulating lipid metabolism in CRC; however, further research is needed. Cruz-Gil et al. (42) found that miRNA-142, miRNA-544a, and miRNA-19b-1 downregulated the expressions of ACSL1, ACSL4, and SCD. Furthermore, high expression of miRNA-19b-1 was related to a better prognosis in patients with stage II and stage III CRC. One study found that miRNA-146-5p was upregulated in CRC, targeting the 3′ UTR mRNA of HOXC10, which stimulated WAT browning and cachexia, maintaining the hypermetabolic state of CRC (43). In addition, cholesterol accumulation is also a feature of altered lipid metabolism in CRC. Sharma et al. (56) found that miR-18a-5p, miR-144-3p, and miR-663b play roles in regulating cholesterol homeostasis in CRC.

### 1.4 Amino acid metabolism

Glutamine is one of the important molecules in tumor metabolic reprogramming; its metabolite α-ketoglutarate (α-KG) is the key metabolic intermediate in the TCA cycle. Alanine, serine, and cysteine transporter 2 (ASCT2), also known as SLC1A5, is an amino acid transporter that is upregulated in many cancers, including KRAS-mutant CRC (57). ASCT2 can transport glutamine into the cytoplasm for catabolism. Glutamine is deaminated by the catalysis of glutaminase (GLS) to generate glutamate, which is transferred to mitochondria and converted to α-KG. α-KG accesses the TCA cycle to provide energy and participates in the synthesis of amino acids.

MiRNAs can affect glutamine metabolism by regulating glutamine transporters and metabolically related enzymes. MiRNA-137 could downregulate ASCT2 and GLS1, reduce glutamine uptake by tumor tissues, and inhibit glutamine metabolism (27, 28). SLC38A1 is a sodium-dependent amino acid transporter that was found to accelerate CRC cell proliferation and metastasis and promote glutamine metabolism while inhibiting apoptosis. However, when miRNA-485-5p was overexpressed, the expression of SLC38A1 was inhibited, and the malignant progression of CRC cells was prevented (36).

## 2 MicroRNAs and signaling pathways

The occurrence of CRC is typically accompanied by the dysregulation of signaling pathways. Multiple signaling pathways jointly induce the occurrence, metabolic disturbance, chemotherapy resistance, and metastasis of CRC. Some miRNAs can regulate this process.

### 2.1 Wnt/β-catenin pathways

**Figure 3:**
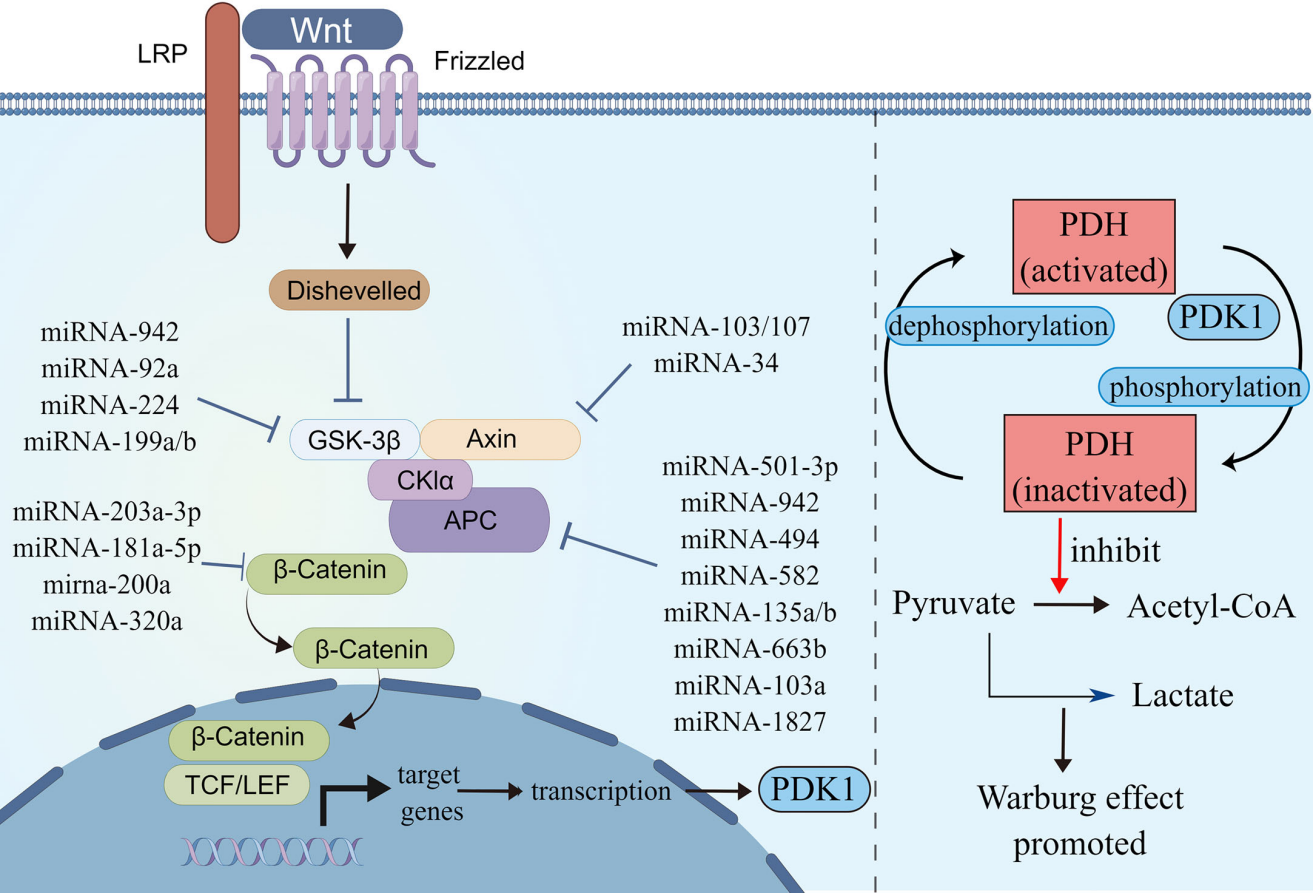
MicroRNAs involved in Wnt/β-catenin signaling pathways. Schematic illustration shows microRNAs that target Wnt/β-catenin signaling pathways (left) and Wnt/β-catenin signaling pathways regulated Warburg effect via PDK1 (right). Pyruvate dehydrogenase (PDH) catalyzes the oxidative decarboxylation of pyruvate. Phosphorylation of PDH by pyruvate dehydrogenase kinase (PDK) results in inactivation and thereby downregulates aerobic respiration and inhibits the formation of acetyl-coenzyme A from pyruvate. As a result, lactic acid production was increased, and Warburg effect was enhanced. The illustration was generated by Figdraw.

The Wnt/β-catenin pathways regulate pluripotent stem cell differentiation, organ development and regeneration, and epithelial mesenchymal transformation (EMT) (58). The dysregulation of the Wnt/β-catenin pathways is the prevalent feature of CRC, which facilitates tumor growth, differentiation, and metabolism in early-stage CRC developments (59). More than 90% of CRC patients have Wnt/β-catenin pathway-related gene mutations, among which APC or CTNNB1 mutations lead to the abnormal activation of the pathway (60). In the absence of the activation of the Wnt/β-catenin signaling pathways, β-catenin is degraded by a protein complex composed of adenomatous polyposis coli (APC), axis inhibitory protein (AXIN), glycogen synthase kinase 3 (GSK3), and casein kinase 1 (CK1), which make β-catenin cannot accumulate in the cytoplasm (61). The activated Wnt/β-catenin signaling pathway inhibits the decomposition of β-catenin. The accumulated β-catenin initiates the transcription of downstream target genes, leading to tumor proliferation and metabolic abnormalities in CRC (62). The Wnt/β-catenin signaling pathway activates downstream target genes to enhance aerobic glycolysis and participates in metabolic reprogramming of tumors. *PDK1* is a downstream gene of the Wnt/β-catenin signaling pathway; it is also a key metabolic regulator of glycolysis and inhibits the transformation of pyruvate to acetyl-CoA and promotes lactate production (**Figure 3**) (63).

Several miRNAs take part in regulating the Wnt/β-catenin signaling pathway. Some miRNAs regulate Wnt/β-catenin signaling by affecting β-catenin expression, some target ligands and receptors in the cell membrane or extracellularly, some regulate the expression of the GSK3β/APC/AXIN/CK1α/PP2A protein complex, while others silence the inhibitors (**Table 2**). MiRNA-150-5p, miRNA-520h, and miRNA-214 are upstream molecules of *CTNNB1.* Their expressions are inhibited in CRC, leading to the silencing of the expression of *CTNNB1*, then inhibit the Wnt/β-catenin signaling pathway and CRC progression (64, 65). MiRNA-203a-3p, miRNA-181a-5p, miRNA-200a, and miRNA-320a could inhibit CRC development by affecting the expression of β-catenin. Among these, MiRNA-203a-3p and miRNA-181a-5p can also reduce the chemoresistance of CRC (66–69).

**Table 2:** List of microRNAs involved in Wnt/β-catenin signaling pathways.

MiRNA	Expression in CRC	Target	Description	effect on CRC	Ref
miRNA-150-5p/miRNA-520h	↓	CTNNB1	Down-regulate the expression of the target gene CTNNB1 to inhibit the Wnt /β -catenin pathway	Inhibit proliferation	64
miRNA-214	↓	CTNNB1	Down-regulate the expression of the target gene CTNNB1 to inhibit the Wnt /β -catenin pathway	Inhibit proliferation	65
miRNA-203a-3p	↓	β-catenin	Target β-catenin and inhibit Wnt/β-catenin signaling	Inhibit proliferation and drug resistance	66
miRNA-181a-5p	↓	β-catenin,TCF4	Target β-catenin and inhibit Wnt/β-catenin signaling	Inhibit proliferation and drug resistance	67
mirna-200a	↓	β-catenin	Target β-catenin and inhibit Wnt/β-catenin signaling	Inhibit proliferation	68
miRNA-320a	↓	β-catenin	Target β-catenin and inhibit Wnt/β-catenin signaling	Inhibit proliferation	79
miRNA-501-3p	↑	APC	Down-regulate the expression of APC and activate wnt/β-catenin signaling	Promote proliferation	69
miRNA-942	↑	APC	Down-regulate the expression of APC and activate wnt/β-catenin signaling	Promote proliferation	70
		GSK3β	Target GSK3β and activate Wnt/β-catenin signaling	Promote proliferation and migration	72
miRNA-494	↑	APC	Down-regulate the expression of APC and activate wnt/β-catenin signaling	Promote proliferation	73
miRNA-582	↑	APC	Down-regulate the expression of APC and activate wnt/β-catenin signaling	Promote proliferation and migration	74
miRNA-135a/b	↑	APC	Down-regulate the expression of APC and activate wnt/β-catenin signaling	Promote proliferation	75
miRNA-663b	↑	APC2	Down-regulate the expression of APC2 and activate wnt/β-catenin signaling	Proliferation, migration and invasion	76
miRNA-103a/miRNA-1827	↑	APC,APC2,Wnt3a,β-catenin	Target APC and APC2, Wnt3a and β-catenin were upregulated and Wnt signal was enhanced	Promote proliferation and Inhibit apoptosis	77
miRNA-137	↓	Wnt3a,β-catenin	Down-regulate the expression of Wnt3a and β-catenin	Inhibition of cell cycle	77
miRNA-103/107	↑	Axin2	Down-regulate the expression of Axin2 and prolong Wnt/β-catenin signaling duration	Induce drug resistance and tumor recurrence	78
mirna-34	↑	Axin2	Down-regulate the expression of Axin2 and prolong Wnt/β-catenin signaling duration	Induce drug resistance and tumor recurrence	79
miRNA-92a	↑	GSK3β	Target GSK3β and activate Wnt/β-catenin signaling	Promote proliferation and drug resistance	80
miRNA-224	↑	GSK3β	Target GSK3β and activate Wnt/β-catenin signaling	Promote proliferation and invasion	81
miRNA-34a	↓	WNT1	Down-regulate the expression of WNT1 and inhibit wnt/β-catenin signaling	Inhibit proliferation and metastatic	82

APC is a kind of tumor-inhibiting factor that antagonizes the Wnt/β-catenin signaling pathway. Some miRNAs were upregulated in CRC and activated the Wnt/β-catenin signaling pathway by affecting the expression of APC or APC2 genes. Previous studies have showed that miRNA-501-3p, miRNA-942, miRNA-494, miRNA-582, and miRNA-135a/b are negatively correlated with APC expression, which induces activation of the Wnt/β-catenin signaling pathway and accelerates CRC development (70, 71, 73–75). Similarly, miRNA-663b activates the Wnt/β-catenin signaling pathway by inhibiting APC2 expression (76). In addition, miRNA-103a and miRNA-1827 are highly expressed in CRC; they downregulate the expression of APC/APC2, upregulate the expression of Wnt3a and β-catenin, and then Wnt/β-catenin signaling is enhanced (77). On the contrary, miRNA-137 downregulated in CRC, but it can inhibit the expression of Wnt3a and β-catenin and lead to the reduction in Wnt/β-catenin signaling. The first two promote CRC proliferation and the last one inhibits it by modulating Wnt/β-catenin signaling pathways.

The AXIN2 gene takes part in degrading β-catenin by reducing the stability of β-catenin. MiRNA-103, miRNA-107, and miRNA-34 could accumulate β-catenin intracellular to induce persistent activation of the Wnt/β-catenin signaling by inhibiting AXIN2, leading to poor prognosis in CRC, including recurrence and chemoresistance (78, 79). Similar to AXIN2, GSK3β is also a tumor-inhibiting factor. Studies have showed that miRNA-92a, miRNA-224, and miRNA-942 inhibit GSK3β, activate Wnt/β-catenin signaling, and accelerate CRC development (72, 80, 81).

A few miRNAs act on cell membranes or extracellular elements, indirectly affecting the Wnt/β-catenin signaling pathway. Li et al. (83) found that miRNA-135b-5p downregulates ZNRF3, then activates the Wnt/β-catenin signaling pathway, leading to invasion and metastasis of CRC. Si et al. (84) revealed that miRNA-1246 is highly expressed in CRC and activates the Wnt/β-catenin signaling pathway to promote migration of CRC. Sun et al. (82) found that miRNA-34a binds to the 3′ UTR of WNT1 and inhibits the expression of WNT1.

### 2.2 EGFR signaling pathways

**Figure 4:**
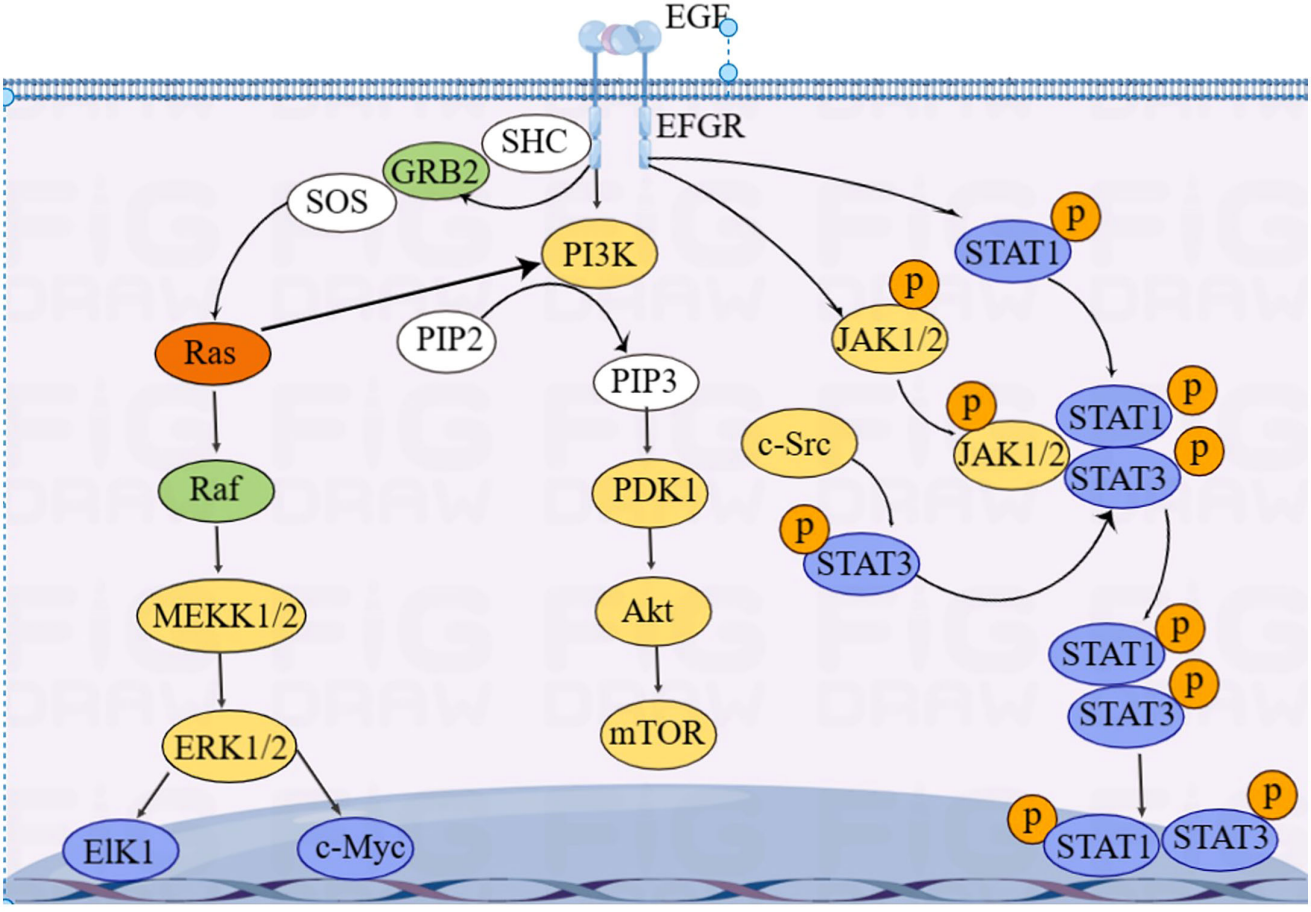
Overview of EGFR signaling pathways.

EGFR is essentially a transmembrane glycoprotein that is located on the surface of epithelial cells and is involved in gene regulation of cell proliferation, differentiation, and apoptosis. EGFR is upregulated in various tumors and is closely correlated with development, metastasis, and chemotherapy resistance of CRC. EGFR is activated to regulate downstream RAS/RAF/MAPK, PI3K/Akt, and JAK/STAT pathways, which transmit signals from the cytoplasm to the nucleus, perform gene regulation, and play a role in promoting tumor cell proliferation and anti-apoptosis (85) (**Figure 4**).

KRAS is an essential molecule in the EGFR signaling pathway. Approximately 40% of CRC cases have KRAS mutations (86). Mutated KRAS does not require ligands to bind to EGFR receptors and directly activate downstream signaling pathways, leading to resistance to anti-EGFR-targeted drugs, such as cetuximab and panitumumab (87). Activation of EGFR signaling pathways increases GLUT1 expression in CRC epithelial cells, inducing the Warburg effect (88).

Du et al. (89) found that miRNA-139-5p is significantly reduced in mutated KRAS CRC and target CTNNB1 and disheveled segment polarity protein 1 (DVL1) to regulate the Wnt/β-catenin signaling pathway. Low expression of miRNA-139-5p is correlated with poor prognosis of CRC. Some studies showed that miRNAs, including let-7, miRNA-16, miRNA-30a, miRNA-30b, miRNA-143/145, miRNA-384, miRNA-622, and miRNA-944, can inhibit KRAS expression, thereby inhibiting CRC invasion and metastasis (90–97).

Some others have also found that mutated KRAS activates the PI3K–AKT–mTOR pathway, leading to metabolic reprogramming in CRC (98, 99). In addition, Xu et al. (100) reported that circRNA_0000392 regulates the PI3K–AKT pathway through sponge miRNA-193a-5p; overexpression of miRNA-193a-5p can significantly reduce the expression level of PIK3R3. Jiang et al. (101) found that circIL4R activates the PI3K–AKT signaling pathway through sponge miR-761. Tang et al. (102) found that miRNA-19a target the PI3K–AKT–mTOR signaling pathway; overexpressed miRNA-19a activates the PI3K–AKT–mTOR signaling pathway and affects the development of CRC.

The activation of STAT3 in the JAK/STAT pathway, a oncogenic transcription factor, is often associated with CRC progression and poor prognosis. Wang et al. (103) found that STAT3 upregulated miRNA-572 expression in CRC cell lines. Modulator of apoptosis 1 (MOAP-1) is a pro-apoptotic protein, and the expression of MOAP-1 is inhibited by miRNA-572. STAT3 induced CRC cell growth, migration, and invasion through miR-572-MOAP-1 pathway. In addition, downregulation of PIAS3, an inhibitor of STAT, and high activation of NF-κB and STAT3 have been observed in CRC patients. Ma et al. (104) found that the activation of STAT3 and NF-κB led to a significant increase in miRNA-18a levels in the colon epithelium, and that overexpressed miRNA-18a promoted CRC growth by inhibiting PIAS3.

### 2.3 TGF-β signaling pathway

**Figure 5:**
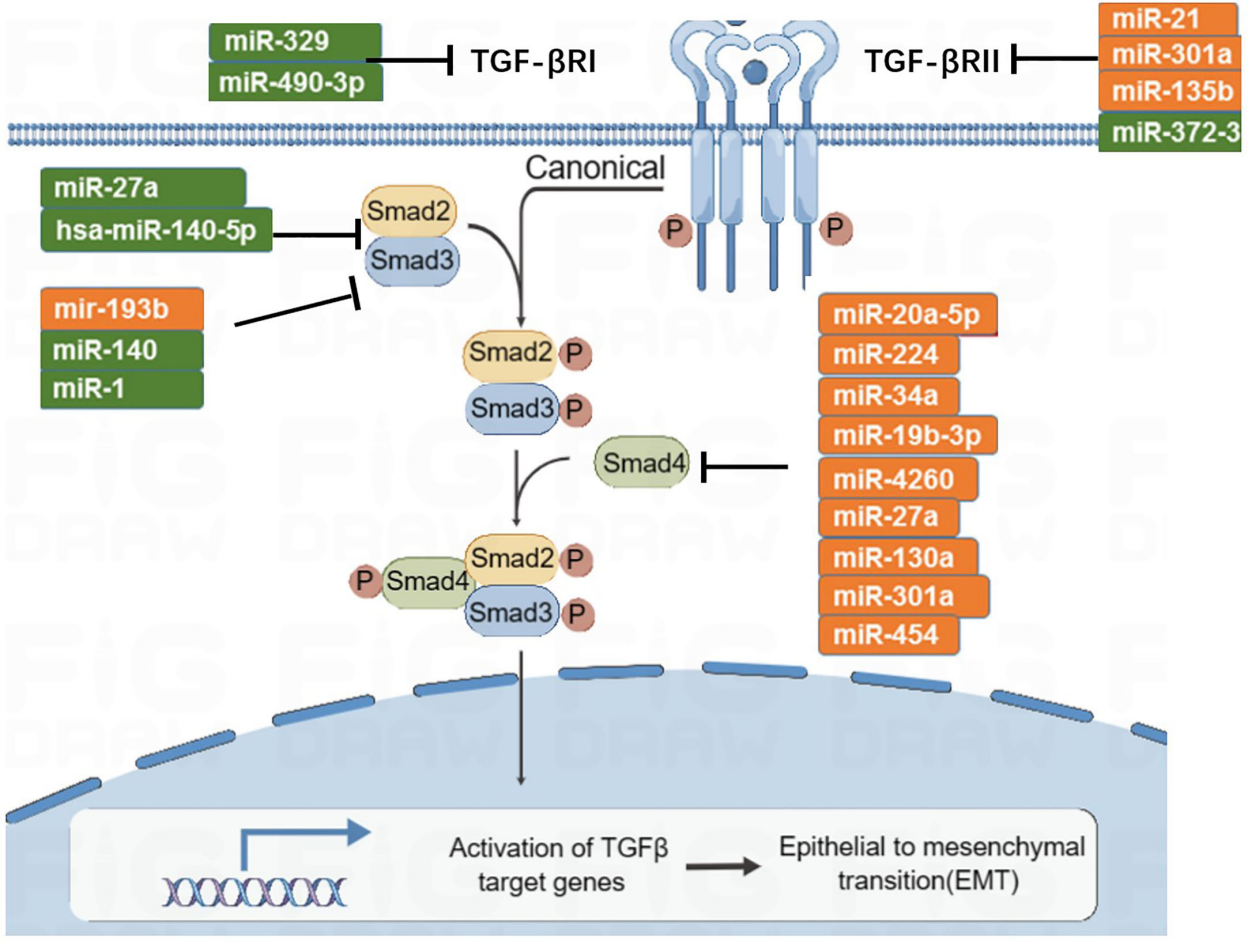
MicroRNAs involved in TGF-β signaling pathway.

The TGF-β signaling pathway has the function of immune supervision. Its main components are TGF-β r1, TGF-β r2, and downstream SMAD molecules (**Figure 5**). TGF-β ligands bind to receptors on the cell membrane, activate downstream SMAD, induce SMAD accumulation in the nucleus, and participate in transcriptional regulation. In the early stage of tumorigenesis, the TGF-β signaling pathway can inhibit tumor proliferation, while in the late stage of the tumor, a persistently elevated or abnormally transduced TGF-β signaling pathway may lead to tumor proliferation and metastasis (105). There are eight subtypes of SMAD. MiRNA-27a targets and inhibits SMAD2 and SMAD4, then inhibits the development and metastasis of CRC (106, 107). Zhai et al. (108) found that miRNA-140-5p is low expressed in CRC; when miRNA-140-5p is overexpressed, it can inhibit the expression of SMAD, resulting in reduced tumor proliferation and invasion. In addition, miRNA-34a and miRNA-18 are negatively correlated with the expression of SMAD4 (109, 110). SMAD6 and SMAD7 are inhibitory molecules that inhibit TGF-β signaling. MiRNA-581 targets SMAD7 and negatively correlates with its expression level. Similarly, miRNA-4775 negatively correlates with the expression level of SMAD7 (111). Both miRNA-581 and miRNA-4775 can promote the invasion and migration of CRC through the SMAD7–TGF-β pathway and induce the epithelial–mesenchymal transformation (EMT) of CRC. In addition, Bu et al. (112) found that miRNA-1269a activates the TGF-β signaling pathway by inhibiting SMAD7, which, in turn, upregulates the expression level of miRNA-1269a, forming a positive feedback loop, thereby promoting the recurrence and metastasis of CRC.

### 2.4 NOTCH pathway

Notch signaling pathway regulates cell proliferation, differentiation, apoptosis, enhanced stem-like properties, and chemoresistance in CRC cells. Notch signaling pathway consists of four parts: Notch receptor, Notch ligand, CSLDNA-binding protein, and the downstream target gene. Notch receptor binding with ligand triggers the activation of Notch signal. Notch receptor proteolysis occurs twice and is transferred to the nucleus, thus activating target gene transcription and regulating protein expression. There are four Notch receptors (Notch1, 2, 3, and 4) and five Notch ligands (Jagged 1, Jagged 2, Delta 1, Delta 3, and Delta 4) in the human body.

MiRNA-34a negatively regulates Notch1 and is downregulated in multiple cancers, including CRC. Zhang et al. (113) found that miRNA-34a could predict patients’ chemotherapy response to 5-FU and was promising in combination with 5-FU for CRC. MiRNA-195-5p inhibits the expression of Notch2 and recombination signal binding protein for immunoglobulin kappa J region (RBPJ) by targeting and binding to their 3′ UTRs, thereby inhibiting cell stemness and chemotherapy resistance of CRC (114). Upregulation of miRNA-206 inhibited the expression of NOTCH3 in SW480 and SW620 cells, leading to the inhibition of CRC proliferation and migration and activation of apoptosis (115). Chen et al. (116) found that miRNA-598 inhibited metastasis in CRC by inactivating the JAG1/Notch2 pathway to inhibit the EMT of CRC cells.

## 3 Clinical diagnosis and treatment strategies based on miRNAs

### 3.1 Early diagnosis of CRC

Patients with early-stage CRC detected through screening have more opportunities for treatment and a better prognosis. Although colonoscopy is the gold standard for CRC diagnosis, it is not widely used owing to its invasiveness and high cost. Some miRNAs are maladjusted in CRC and can serve as biomarkers for diagnosis. A meta-analysis showed that serum miRNA-21 had a sensitivity and specificity of 77% and 83%, respectively, for CRC, showing its potential diagnostic value (117). Furthermore, miRNA-92a, miRNA-18a, miRNA-144, and miRNA-29a have been studied and showed a good sensitivity and specificity, thus indicating that they are possible biomarkers for CRC diagnosis (118–121). Some miRNAs derived from the serum or stool may be used as novel biomarkers for screening assays.

Yau et al. (122) found that a fecal immunochemistry test (FIT), combined with miRNA-21 and miRNA-92a, could improve the accuracy of detections. In addition, some miRNA panels have high diagnostic efficacy and research value, including miRNA-15b,miRNA-21, and miRNA-31 (sensitivity, 0.95; specificity, 0.94; AUC, 0.948); miRNA-144-3p, miRNA-425-5p, and miRNA-1260b (sensitivity, 0.93; specificity, 0.91; AUC, 0.954); and miRNA-1246, miRNA-202-3p, miRNA-21-3p, miRNA-1229-3p, and miRNA-532-3p (sensitivity, 0.91; specificity, 0.91; AUC, 0.96) (123). These findings demonstrate the potential of miRNAs for early CRC screening test.

### 3.2 Prognosis of CRC

The 5-year relative survival of CRC remains at only 68%. The main reasons for the poor prognosis of patients with advanced CRC are tumor metastasis and recurrence. Approximately 50% of advanced CRC will metastasize, accounting for the high mortality rate of CRC patients. After radical surgery, approximately a third of patients experience tumor recurrence (124). Therefore, it is necessary to develop new therapeutic modalities and prognostic biomarkers. MiRNAs are involved in tumorigenesis, progression, and metastasis (125). Many researchers have found various miRNAs as prognostic biomarkers and therapeutic targets for CRC.

#### 3.2.1 Metastasis of CRC

EMT is a biological process where epithelial cells derived from malignant tumor cells acquire the ability to migrate and invade. EMT is related to the metastasis of CRC. Many miRNAs interact directly or indirectly with EMT-related molecules to induce or inhibit the progression of EMTs, thereby affecting tumor metastasis. Therefore, prevention of EMT may be an effective method to inhibit tumor metastasis.

SNAIL, ZEB1, and ZEB2 are transcription factors of EMT that regulate epithelial and mesenchymal markers and induce EMT in CRC. Members of the miRNA-200 family (miRNA-200a, miRNA-200b, miRNA-200c, miRNA-141, and miRNA-429) reduce the migration and invasion of CRC cells by inhibiting ZEB1 and ZEB2 mRNA translation. MiRNA-132, miRNA-92, and miRNA-335 also directly target ZEB2. Members of the miRNA-34 family suppress SNAIL and inhibit EMT, reducing the migration of CRC cells. Downregulation of these miRNAs is associated with distant metastasis and advanced tumors (126). In addition, miRNA-4775 and miRNA-496 promote EMT and migration in CRC through the TGF-β and WNT/β signaling pathways, respectively (127, 128). miRNA-192 and miRNA-194, which are downstream molecules of SNAIL, inhibit EMT in CRC (129). MiRNA-612, miRNA-219-5p, miRNA-185, miRNA-296, and miRNA-421 target carcinogenic factors and inhibit metastasis of EMT and CRC (130).

Another characteristic of tumor metastasis is angiogenesis, a process that provides the necessary oxygen and nutrients for tumor growth and metastasis. Vascular endothelial growth factor (VEGF) and hypoxia-inducible factor 1 (HIVE-1) regulate the formation of blood vessels (131). MiRNA-590-5p, miRNA-1249, and miRNA-622 inhibit CRC angiogenesis and metastasis by regulating VEGF-A (132–134). MiRNA-2A is a downstream factor of VEGF-A, and its overexpression suppresses VEGF-A expression and inhibits CRC angiogenesis (135). MiRNA-206 and miRNA-107 regulate hypoxia signal transduction and inhibit angiogenesis by suppressing HIF1α and HIF1β, respectively (136, 137).

Metastasis of colorectal cancer is a complex process that involves EMT, angiogenesis, and remodeling of TME. Prevention of these processes is an effective strategy to inhibit tumor metastasis, and miRNAs are involved in the regulation of these processes. Therefore, miRNAs can serve as biomarkers to determine the risk of CRC metastasis.

#### 3.2.2 Recurrence of CRC

The 5-year overall recurrence rate was 9.3% for stage I CRC, 27.2% for Stage II CRC, and 56.1% for Stage III CRC. Tumor TMN stage is considered a main risk factor and the only independent predictor of recurrence (138). However, recently, miRNAs have been demonstrated as potential biomarkers to predict CRC recurrence.

For example, Kandimalla et al. (139) developed an miRNA-recurrence classifier (MRC) by identifying an eight-miRNA signature based on three independent genome-wide miRNA-expression profiling datasets. By comparing the miRNA expression profiles of high- and low-risk Stage II and III CRC patients, they identified eight miRNAs that are most statistically significant: hImir-191,Ia-mir-2I, hsa-miI0b, hsaIr-30c2,Ia-mir-33a, I-mir-362, hsa-mir-429, and hsa-mir-744. After validation, these MRCS identified high-risk CRC patients and predicted CRC recurrence. In the two validation cohorts, the AUC values for predicting tumor recurrence were 0.79 (95%CI, 0.67–0.89) and 0.88 (95%CI, 0.78-0.99), respectively.

Fukada et al. (140) collected plasma samples from 103 CRC patients at four different periods and analyzed postoperative plasma mirNA-5P levels. They concluded that postoperative plasma miRNA-21-5p levels can effectively predict recurrence and progressive disease. Yuan et al. (141) found that postoperative plasma miRNA-31, miRNA-141, and miRNA-16 could predict the recurrence of CRC. Moreover, patients with high miRNA-19a expression had a significantly lower disease-free survival than those with low miRNA-19a expression, suggesting that miRNA-19a may be used to predict CRC recurrence (142).

### 3.3 Chemotherapy resistance of CRC

Surgery is the most effective treatment for CRC; however, when the patient is in the advanced stage of the disease, surgical resection alone does not significantly prolong overall survival. In these cases, adjuvant therapy, such as chemotherapy, radiotherapy, and targeted drug therapy, can effectively reduce tumor volume and prolong patient survival. Chemotherapy is an effective treatment for most CRC patients, but resistance to chemotherapy can reduce its effectiveness. The miRNA expression of patients receiving chemotherapy differ significantly. First-line chemotherapy drugs for CRC include 5-FU, capecitabine, oxaliplatin, irinotecan, cetuximab, and bevacizumab. miRNA levels can affect the targets of chemotherapeutic drugs or related signaling pathways. Therefore, the miRNA level of patients’ serum can be used to predict the efficacy of chemotherapy and provide a reference for clinical decision-making. These mechanisms can guide clinicians in choosing the most appropriate chemotherapy regimen.

#### 3.3.1 Capecitabine and 5-FU

Capecitabine is an oral fluorouracil that is converted to 5-FU in tumor tissues, and 5-FU inhibits tumor growth by interfering with DNA synthesis. MiRNAs can regulate the transduction of signaling pathways to regulate chemotherapy resistance in tumors. MiRNA-149 and miRNA-320 target FoxM1 and affect the Wnt/β-catenin signaling pathway and glucose metabolism, thereby increasing the sensitivity of 5-FU (143, 144). MiRNA-135b, miRNA-182, and miRNA-587 can promote 5-FU resistance in CRC by activating the PI3K–AKT pathway (145, 146). In contrast, miRNA-302 and miRNA-20b inhibit the EGFR–AKT pathway and increase the sensitivity of 5-FU in CRC (147, 148).

#### 3.3.2 Oxaliplatin

Oxaliplatin acts on DNA and inhibits tumor growth and proliferation by blocking DNA replication and transcription. It is often used in combination with other anticancer agents. The FOLFOX (5-FU combined with oxaliplatin) and CapeOX (capecitabine combined with oxaliplatin) regimens showed better response rates, progression-free survival, and overall survival in first-line treatment of advanced or metastatic CRC. Activation of the Wnt/β-catenin signaling pathway is correlated with oxaliplatin resistance in CRC. MiRNA-103 and miRNA-107 promote the Wnt/β-catenin signaling pathway to induce oxaliplatin resistance in CRC (74). In addition, miRNA-506 overexpression inhibits the Wnt/β-catenin signaling pathway and enhances sensitivity to oxaliplatin (149). In the EGFR signaling pathway, miRNA-17 and miRNA-19a can downregulate PTEN and reduce oxaliplatin resistance in CRC (150, 151). Furthermore, Zhou et al. (152) found that miRNA-203 induced oxaliplatin resistance in CRC by inhibiting ataxia telangiectasia mutated (ATM) kinase expression.

#### 3.3.3 Irinotecan

The FOLFIRI regimen (irinotecan combined with 5-FU) is not suitable for postoperative adjuvant chemotherapy for CRC but can be used for palliative treatment of advanced or metastatic CRC. Bitarte et al. (153) found that miRNA-451 expression levels were lower in patients who did not respond to first-line irinotecan therapy, which was associated with downregulation of miRNA-451 expression in the intestinal nodal globule. The expression of miRNA-451 led to the self-renewal of intestinal nodal globules, reducing their oncogenicity and resistance to irinotecan chemotherapy. In addition, the overexpression of miRNA-146a, miRNA-194, miRNA-514b-3p, and miRNA-519c can increase the sensitivity of CRC to irinotecan (154–157).

#### 3.3.4 Cetuximab

Cetuximab is an EGFR-targeting monoclonal antibody commonly used in wild-type KRAS advanced or metastatic CRC patients. The upregulation of miRNA-100/125b promoted Wnt/β-catenin signaling and mediated resistance to cetuximab in CRC (158).

### 3.4 Treatment of CRC based on miRNAs

In addition to the increased sensitivity of chemotherapeutic agents mentioned above, there are two miRNAs-based therapies: (1) miRNAs used in immunotherapy (2) miRNAs as therapeutic targets.

There are approximately 15% of tumors with microsatellite instability-high (MSI-H) in CRC. PD-1 and PD-L1 are immune checkpoint molecules of MSI-H CRC, which is resistant to the antitumor immune response. Therefore, anti-PD-1 therapy, a kind of immune-checkpoint inhibitor (ICI) therapy, has greatly improved the efficiency of MSI-H CRC treatment. MiRNAs can target this immune checkpoint to play a role in tumor immunotherapy. Liu et al. (159) found that miRNA-15b-5p suppressed the expression of PD-L1, inhibited tumor progression, and increased the sensitivity to anti-PD-1 drugs. Ashizawa et al. (160) found that miR-148a-3p directly binds to the 3′ UTR of PD-L1, thereby reducing whole-cell and cell-surface PD-L1 expression in HCT116 and SW837 cell lines. In addition, miR-140-3p, miR-382-3p, miR-148a-3p, miR-93-5p, miR-200a-3p, miR-200c-3p, miR-138-5p, and miR-15b-5p can substantially reduce tumor migration, inhibit tumor development, stimulate anti-tumoral immune responses, decrease tumor viability, and enhance the chemosensitivity of colorectal cancer cells by inhibiting PD-L1 (161).

Tumor-related miRNAs can be divided into two categories: oncomiRNAs are overexpressed in tumors, whereas tumor-suppressor miRNAs are underexpressed. Two treatment strategies have been proposed for treating patients with cancer using miRNA-based therapy: suppression of oncomiRNAs and upregulation of tumor suppressor miRNAs (162). These methods involve the artificially regulation of miRNA expression. The former usually involves the use of miRNA mimics to artificially increase the expression of target miRNA; hence, that it can exert tumor inhibition. Meanwhile, the latter can be achieved using miRNA antagonists, including antisense oligonucleotides, antagomirs, and miRNA sponges. Wu et al. (163) developed a novel miRNA-129 mimic (Mimic-1) that can be delivered to cancer cells without the need for any delivery vehicle. Mimic-1 showed potent efficacy in eliminating resistant colon cancer stem cells both *in vitro* and *in vivo*.

Although miRNAs have shown great potential in the diagnosis, prognosis, and treatment of CRC, their application in clinical practice requires more extensive and in-depth research.

## 4 Conclusion and prospective

MiRNAs are involved in almost all pathophysiological processes in CRC and play an essential role in its occurrence and development, metabolic reprogramming, abnormal signaling pathways, treatment, and prognosis. The changes in some miRNAs, in combination with conventional detection methods, can improve the diagnosis of CRC at an early stage. The expression levels of some miRNAs can also predict the efficacy of chemotherapy and help choose the best chemotherapy regimen. Therapeutic strategies that target the correction of maladjusted miRNAs are promising. In the future, we can develop screening assays with better sensitivity and specificity based on relevant miRNAs, suppress tumor growth by correcting abnormally elevated or suppressed miRNA expression, predict chemotherapy efficacy based on miRNA expression, and synergistically treat tumors using chemotherapy drugs. Our understanding of some miRNAs is not perfect, clinical translation of theoretical knowledge remains limited, and further research is needed. Investigating the role of miRNAs in the occurrence and treatment of CRC is of great significance for improving its diagnosis and prognosis.

## Author contributions

All authors have read and agreed to the published version of the manuscript. BX and QH drafted the first version of this manuscript. HZ collected literature. SL provided critical revisions and edited the manuscript. JX revised the manuscript. All authors contributed to the article and approved the submitted version.

## Glossary

**Table:**

CRC	colorectal cancer; miRNA, microRNA; DFS, disease-free survival
GLUT	glucose transporter
SLC2A1	solute carrier family 2 member 1
HK	hexokinase
PFK	phosphofructokinase
G-6-P	glucose-6-phosphate
F-6-P	fructose-6-phosphate
F-1, 6-P_2_	fructose-1, 6-bisphosphate
PFKFB3	6-phosphofructo-2-kinase/fructose-2,6-biphosphatase 3
PEP	phosphoenolpyruvate
PK	pyruvate kinase
LDH	lactate dehydrogenase
FA-CoA	fatty acid CoA
ACS	acyl-CoA synthase
SCD	stearoyl-CoA desaturase
ASCT2	alanine, serine, cysteine transporter 2
GLS	glutaminase
α-KG	α -ketoglutaric acid
TCA	tricarboxylic acid
CTNNB1	catenin beta 1
APC	adenomatous polyposis coli
AXIN	axis inhibition protein
CK1	casein kinase 1
GSK3	glycogen synthase kinase 3
PP2A	protein phosphatase 2A
DVL1	disheveled segment polarity protein 1
AKT	protein kinase B
EGFR	epidermal growth factor receptor
FIT	fecal immunochemistry test
PDHX	pyruvate dehydrogenase protein X component
CKS1B	CDC28 protein kinase regulatory subunit 1B
SDHB	succinate dehydrogenase-B
PDK1	pyruvate dehydrogenase kinase 1
FOXM1	forkhead box M1
FU	fluorouracil

## References

[1] Sung H Ferlay J Siegel RL Laversanne M Soerjomataram I Jemal A. Global cancer statistics 2020: GLOBOCAN estimates of incidence and mortality worldwide for 36 cancers in 185 countries. CA Cancer J Clin (2021) 71:209–49. doi: 10.3322/caac.21660 33538338

[2] Shaukat A Kahi CJ Burke CA Rabeneck L Sauer BG Rex DK. ACG clinical guidelines: colorectal cancer screening 2021. Am J Gastroenterol (2021) 116:458–79. doi: 10.14309/ajg.0000000000001122 33657038

[3] Patel SG Murphy CC Lieu CH Hampel H. Early age onset colorectal cancer. Adv Cancer Res (2021) 151:1–37. doi: 10.1016/bs.acr.2021.03.001 34148611

[4] Song M Chan AT. Environmental factors, gut microbiota, and colorectal cancer prevention. Clin Gastroenterol Hepatol Off Clin Pract J Am Gastroenterological Assoc (2019) 17:275–89. doi: 10.1016/j.cgh.2018.07.012 PMC6314893 30031175

[5] DAS FC Wernhoff P Dominguez-Barrera C Dominguez-Valentin M. Update on hereditary colorectal cancer. Anticancer Res (2016) 36:4399–405. doi: 10.21873/anticanres.10983 27630275

[6] Pavlova NN Thompson CB. The emerging hallmarks of cancer metabolism. Cell Metab (2016) 23:27–47. doi: 10.1016/j.cmet.2015.12.006 26771115 PMC4715268

[7] Esteva M Leiva A Ramos M Pita-Fernandez S Gonzalez-Lujan L Casamitjana M. Factors related with symptom duration until diagnosis and treatment of symptomatic colorectal cancer. BMC Canc (2013) 13:87. doi: 10.1186/1471-2407-13-87 PMC3598975 23432789

[8] Zhang Y Chen Z Li J. The current status of treatment for colorectal cancer in China: a systematic review. Medicine (2017) 96:e8242. doi: 10.1097/MD.0000000000008242 28984783 PMC5738019

[9] Ludmir EB Palta M Willett CG Czito BG. Total neoadjuvant therapy for rectal cancer: an emerging option. Cancer (2017) 123:1497–506. doi: 10.1002/cncr.30600 28295220

[10] Lee RC Feinbaum RL Ambros V. The c. elegans heterochronic gene lin-4 encodes small RNAs with antisense complementarity to lin-14. Cell (1993) 75:843–54. doi: 10.1016/0092-8674(93)90529-Y 8252621

[11] Hibner G Kimsa-Furdzik M Francuz T. Relevance of MicroRNAs as potential diagnostic and prognostic markers in colorectal cancer. Int J Mol Sci (2018) 19. doi: 10.3390/ijms19102944 PMC6213499 30262723

[12] Lewis BP Burge CB Bartel DP. Conserved seed pairing, often flanked by adenosines, indicates that thousands of human genes are microRNA targets. Cell (2005) 120:15–20. doi: 10.1016/j.cell.2004.12.035 15652477

[13] Alyami NM. MicroRNAs role in breast cancer: theranostic application in Saudi Arabia. Front Oncol (2021) 11:717759. doi: 10.3389/fonc.2021.717759 34760689 PMC8573223

[14] Zhao J Chen Y Liu F Yin M. Overexpression of miRNA-143 inhibits colon cancer cell proliferation by inhibiting glucose uptake. Arch Med Res (2018) 49:497–503. doi: 10.1016/j.arcmed.2018.12.009 30595365

[15] Gregersen LH Jacobsen A Frankel LB Wen J Krogh A Lund AH. MicroRNA-143 down-regulates hexokinase 2 in colon cancer cells. BMC Canc (2012) 12:232. doi: 10.1186/1471-2407-12-232 PMC3480834 22691140

[16] Santasusagna S Moreno I Navarro A Munoz C Martinez F Hernandez R. miR-328 mediates a metabolic shift in colon cancer cells by targeting SLC2A1/GLUT1. Clin Trans Oncol Off Publ Fed Spanish Oncol Societies Natl Cancer Institute Mexico (2018) 20:1161–7. doi: 10.1007/s12094-018-1836-1 PMC6105238 29374351

[17] Zhang ZJ Zhang YH Qin XJ Wang YX Fu J. Circular RNA circDENND4C facilitates proliferation, migration and glycolysis of colorectal cancer cells through miR-760/GLUT1 axis. Eur Rev Med Pharmacol Sci (2020) 24:2387–400. 10.26355/eurrev_202003_20506 32196590

[18] Li S Zhu K Liu L Gu J Niu H Guo J. lncARSR sponges miR-34a-5p to promote colorectal cancer invasion and metastasis via hexokinase-1-mediated glycolysis. Cancer science (2020) 111:3938–52. doi: 10.1111/cas.14617 PMC7540992 32798250

[19] Qin Y Cheng C Lu H Wang Y. miR-4458 suppresses glycolysis and lactate production by directly targeting hexokinase2 in colon cancer cells. Biochem Biophys Res Commun (2016) 469:37–43. doi: 10.1016/j.bbrc.2015.11.066 26607110

[20] Liu Y Li H Ye X Ji A Fu X Wu H. Hsa_circ_0000231 knockdown inhibits the glycolysis and progression of colorectal cancer cells by regulating miR-502-5p/MYO6 axis. World J Surg Oncol (2020) 18:255. doi: 10.1186/s12957-020-02033-0 32993655 PMC7526375

[21] Li C Yu Z Ye J. MicroRNA-513a-3p regulates colorectal cancer cell metabolism via targeting hexokinase 2. Exp Ther Med (2020) 20:572–80. doi: 10.3892/etm.2020.8727 PMC7282190 32537015

[22] Zhu W Huang Y Pan Q Xiang P Xie N Yu H. MicroRNA-98 suppress warburg effect by targeting HK2 in colon cancer cells. Digestive Dis Sci (2017) 62:660–8. doi: 10.1007/s10620-016-4418-5 28025745

[23] Cui S Yang X Zhang L Zhao Y Yan W. LncRNA MAFG-AS1 promotes the progression of colorectal cancer by sponging miR-147b and activation of NDUFA4. Biochem Biophys Res Commun (2018) 506:251–8. doi: 10.1016/j.bbrc.2018.10.112 30348529

[24] Deng X Li D Ke X Wang Q Yan S Xue Y. Mir-488 alleviates chemoresistance and glycolysis of colorectal cancer by targeting PFKFB3. J Clin Lab Anal (2021) 35:e23578. doi: 10.1002/jcla.23578 32990355 PMC7843269

[25] Ren J Li W Pan G Huang F Yang J Zhang H. miR-142-3p modulates cell invasion and migration via PKM2-mediated aerobic glycolysis in colorectal cancer. Analytical Cell Pathol (Amsterdam). (2021) 2021:9927720. doi: 10.1155/2021/9927720 PMC8294993 34336555

[26] Zheng H Zhang M Ke X Deng X Li D Wang Q. LncRNA XIST/miR-137 axis strengthens chemo-resistance and glycolysis of colorectal cancer cells by hindering transformation from PKM2 to PKM1. Cancer Biomarkers section A Dis markers (2021) 30:395–406. doi: 10.3233/CBM-201740 PMC12499988 33386794

[27] Dong J Xiao D Zhao Z Ren P Li C Hu Y. Epigenetic silencing of microRNA-137 enhances ASCT2 expression and tumor glutamine metabolism. Oncogenesis (2017) 6:e356. doi: 10.1038/oncsis.2017.59 28692032 PMC5541711

[28] Li J Song P Jiang T Dai D Wang H Sun J. Heat shock factor 1 epigenetically stimulates glutaminase-1-Dependent mTOR activation to promote colorectal carcinogenesis. Mol Ther J Am Soc Gene Ther (2018) 26:1828–39. doi: 10.1016/j.ymthe.2018.04.014 PMC6035735 29730197

[29] Taniguchi K Sugito N Kumazaki M Shinohara H Yamada N Nakagawa Y. MicroRNA-124 inhibits cancer cell growth through PTB1/PKM1/PKM2 feedback cascade in colorectal cancer. Cancer Lett (2015) 363:17–27. doi: 10.1016/j.canlet.2015.03.026 25818238

[30] Fu R Yang P Amin S Li Z. A novel miR-206/hnRNPA1/PKM2 axis reshapes the warburg effect to suppress colon cancer growth. Biochem Biophys Res Commun (2020) 531:465–71. doi: 10.1016/j.bbrc.2020.08.019 32800545

[31] Huang X Hou Y Weng X Pang W Hou L Liang Y. Diethyldithiocarbamate-copper complex (CuET) inhibits colorectal cancer progression via miR-16-5p and 15b-5p/ALDH1A3/PKM2 axis-mediated aerobic glycolysis pathway. Oncogenesis. (2021) 10:4. doi: 10.1038/s41389-020-00295-7 33419984 PMC7794448

[32] Wang J Wang H Liu A Fang C Hao J Wang Z. Lactate dehydrogenase a negatively regulated by miRNAs promotes aerobic glycolysis and is increased in colorectal cancer. Oncotarget (2015) 6:19456–68. doi: 10.18632/oncotarget.3318 PMC4637298 26062441

[33] Jiang Z Hu H Hu W Hou Z Liu W Yu Z. Circ-RNF121 regulates tumor progression and glucose metabolism by miR-1224-5p/FOXM1 axis in colorectal cancer. Cancer Cell Int (2021) 21:596. doi: 10.1186/s12935-021-02290-3 34742305 PMC8572430

[34] Zhang Z Yang W Li N Chen X Ma F Yang J. LncRNA MCF2L-AS1 aggravates proliferation, invasion and glycolysis of colorectal cancer cells via the crosstalk with miR-874-3p/FOXM1 signaling axis. Carcinogenesis (2021) 42:263–71. doi: 10.1093/carcin/bgaa093 32860508

[35] Wang X Tao G Huang D Liang S Zheng D. Circular RNA NOX4 promotes the development of colorectal cancer via the microRNA4855p/CKS1B axis. Oncol Rep (2020) 44:2009–20. 10.3892/or.2020.7758 PMC7551031 32901890

[36] Yu J Chen X Li J Wang F. CircRUNX1 functions as an oncogene in colorectal cancer by regulating circRUNX1/miR-485-5p/SLC38A1 axis. Eur J Clin Invest (2021) 51:e13540. doi: 10.1111/eci.13540 33769559

[37] Wei Z Cui L Mei Z Liu M Zhang D. miR-181a mediates metabolic shift in colon cancer cells via the PTEN/AKT pathway. FEBS Lett (2014) 588:1773–9. doi: 10.1016/j.febslet.2014.03.037 24685694

[38] Guo X Zhu Y Hong X Zhang M Qiu X Wang Z. miR-181d and c-myc-mediated inhibition of CRY2 and FBXL3 reprograms metabolism in colorectal cancer. Cell Death disease (2017) 8:e2958. doi: 10.1038/cddis.2017.300 28749470 PMC5550850

[39] Chen B Liu Y Jin X Lu W Liu J Xia Z. MicroRNA-26a regulates glucose metabolism by direct targeting PDHX in colorectal cancer cells. BMC Canc (2014) 14:443. doi: 10.1186/1471-2407-14-443 PMC4071217 24935220

[40] Liu S Xiao Z Ai F Liu F Chen X Cao K. miR-142-5p promotes development of colorectal cancer through targeting SDHB and facilitating generation of aerobic glycolysis. Biomed pharmacother = Biomed pharmacotherapie (2017) 92:1119–27. doi: 10.1016/j.biopha.2017.05.134 28622713

[41] Gharib E Nasri Nasrabadi P Reza Zali M. miR-497-5p mediates starvation-induced death in colon cancer cells by targeting acyl-CoA synthetase-5 and modulation of lipid metabolism. J Cell Physiol (2020) 235:5570–89. doi: 10.1002/jcp.29488 32012265

[42] Cruz-Gil S Sanchez-Martinez R Gomez de Cedron M Martin-Hernandez R Vargas T Molina S. Targeting the lipid metabolic axis ACSL/SCD in colorectal cancer progression by therapeutic miRNAs: miR-19b-1 role. J Lipid Res (2018) 59:14–24. doi: 10.1194/jlr.M076752 29074607 PMC5748493

[43] Di W Zhang W Zhu B Li X Tang Q Zhou Y. Colorectal cancer prompted adipose tissue browning and cancer cachexia through transferring exosomal miR-146b-5p. J Cell Physiol (2021) 236:5399–410. doi: 10.1002/jcp.30245 33368224

[44] Lu J Tan M Cai Q. The warburg effect in tumor progression: mitochondrial oxidative metabolism as an anti-metastasis mechanism. Cancer Lett (2015) 356:156–64. doi: 10.1016/j.canlet.2014.04.001 PMC4195816 24732809

[45] Lunt SY Vander Heiden MG. Aerobic glycolysis: meeting the metabolic requirements of cell proliferation. Annu Rev Cell Dev Biol (2011) 27:441–64. doi: 10.1146/annurev-cellbio-092910-154237 21985671

[46] Ganapathy V Thangaraju M Prasad PD. Nutrient transporters in cancer: relevance to warburg hypothesis and beyond. Pharmacol Ther (2009) 121:29–40. doi: 10.1016/j.pharmthera.2008.09.005 18992769

[47] Xu S Herschman HR. A tumor agnostic therapeutic strategy for hexokinase 1-Null/Hexokinase 2-positive cancers. Cancer Res (2019) 79:5907–14. doi: 10.1158/0008-5472.CAN-19-1789 PMC12139393 31434645

[48] Uyeda K. Short- and long-term adaptation to altered levels of glucose: fifty years of scientific adventure. Annu Rev Biochem (2021) 90:31–55. doi: 10.1146/annurev-biochem-070820-125228 34153217

[49] Shi L Pan H Liu Z Xie J Han W. Roles of PFKFB3 in cancer. Signal Transduct Target Ther (2017) 2:17044. doi: 10.1038/sigtrans.2017.44 29263928 PMC5701083

[50] Dayton TL Jacks T Vander Heiden MG. PKM2, cancer metabolism, and the road ahead. EMBO Rep (2016) 17:1721–30. doi: 10.15252/embr.201643300 PMC5283597 27856534

[51] Wang X Zhang H Yang H Bai M Ning T Deng T. Exosome-delivered circRNA promotes glycolysis to induce chemoresistance through the miR-122-PKM2 axis in colorectal cancer. Mol Oncol (2020) 14:539–55. doi: 10.1002/1878-0261.12629 PMC7053238 31901148

[52] Li Z Zhang H. Reprogramming of glucose, fatty acid and amino acid metabolism for cancer progression. Cell Mol Life Sci CMLS (2016) 73:377–92. doi: 10.1007/s00018-015-2070-4 PMC11108301 26499846

[53] Zdralevic M Brand A Di Ianni L Dettmer K Reinders J Singer K. Double genetic disruption of lactate dehydrogenases a and b is required to ablate the "Warburg effect" restricting tumor growth to oxidative metabolism. J Biol Chem (2018) 293:15947–61. doi: 10.1074/jbc.RA118.004180 PMC6187639 30158244

[54] Zhang D Yang N. MiR-335-5p inhibits cell proliferation, migration and invasion in colorectal cancer through downregulating LDHB. J BUON Off J Balkan Union Oncol (2019) 24:1128–36. 31424671

[55] Koundouros N Poulogiannis G. Reprogramming of fatty acid metabolism in cancer. Br J Canc (2020) 122:4–22. doi: 10.1038/s41416-019-0650-z PMC6964678 31819192

[56] Sharma B Randhawa V Vaiphei K Gupta V Dahiya D Agnihotri N. Expression of miR-18a-5p, miR-144-3p, and miR-663b in colorectal cancer and their association with cholesterol homeostasis. J Steroid Biochem Mol Biol (2021) 208:105822. doi: 10.1016/j.jsbmb.2021.105822 33465419

[57] Toda K Nishikawa G Iwamoto M Itatani Y Takahashi R Sakai Y. Clinical role of ASCT2 (SLC1A5) in KRAS-mutated colorectal cancer. Int J Mol Sci (2017) 18. doi: 10.3390/ijms18081632 PMC5578022 28749408

[58] Shirmohamadi M Eghbali E Najjary S Mokhtarzadeh A Kojabad AB Hajiasgharzadeh K. Regulatory mechanisms of microRNAs in colorectal cancer and colorectal cancer stem cells. J Cell Physiol (2020) 235:776–89. doi: 10.1002/jcp.29042 31264216

[59] Bian J Dannappel M Wan C Firestein R. Transcriptional regulation of wnt/beta-catenin pathway in colorectal cancer. Cells (2020) 9. doi: 10.3390/cells9092125 PMC7564852 32961708

[60] Cancer Genome Atlas N. Comprehensive molecular characterization of human colon and rectal cancer. Nature (2012) 487:330–7. doi: 10.1038/nature11252 PMC3401966 22810696

[61] Cheng X Xu X Chen D Zhao F Wang W. Therapeutic potential of targeting the wnt/beta-catenin signaling pathway in colorectal cancer. Biomed pharmacother = Biomed pharmacotherapie (2019) 110:473–81. doi: 10.1016/j.biopha.2018.11.082 30530050

[62] Schuijers J Mokry M Hatzis P Cuppen E Clevers H. Wnt-induced transcriptional activation is exclusively mediated by TCF/LEF. EMBO J (2014) 33:146–56. doi: 10.1002/embj.201385358 PMC3989608 24413017

[63] Pate KT Stringari C Sprowl-Tanio S Wang K TeSlaa T Hoverter NP. Wnt signaling directs a metabolic program of glycolysis and angiogenesis in colon cancer. EMBO J (2014) 33:1454–73. doi: 10.15252/embj.201488598 PMC4194089 24825347

[64] Zhou T Wu L Ma N Tang F Zong Z Chen S. LncRNA PART1 regulates colorectal cancer via targeting miR-150-5p/miR-520h/CTNNB1 and activating wnt/beta-catenin pathway. Int J Biochem Cell Biol (2020) 118:105637. doi: 10.1016/j.biocel.2019.105637 31669140

[65] Chandrasekaran KS Sathyanarayanan A Karunagaran D. miR-214 activates TP53 but suppresses the expression of RELA, CTNNB1, and STAT3 in human cervical and colorectal cancer cells. Cell Biochem Funct (2017) 35:464–71. doi: 10.1002/cbf.3304 29023799

[66] Xiao Z Qu Z Chen Z Fang Z Zhou K Huang Z. LncRNA HOTAIR is a prognostic biomarker for the proliferation and chemoresistance of colorectal cancer via MiR-203a-3p-Mediated wnt/ß-catenin signaling pathway. Cell Physiol Biochem Int J Exp Cell physiology biochemistry Pharmacol (2018) 46:1275–85. doi: 10.1159/000489110 29680837

[67] Han P Li JW Zhang BM Lv J-c Li Y-m Gu X-y. The lncRNA CRNDE promotes colorectal cancer cell proliferation and chemoresistance via miR-181a-5p-mediated regulation of wnt/beta-catenin signaling. Mol canc (2017) 16:9. doi: 10.1186/s12943-017-0583-1 PMC5237133 28086904

[68] Yang W Ning N Jin X. The lncRNA H19 promotes cell proliferation by competitively binding to miR-200a and derepressing beta-catenin expression in colorectal cancer. BioMed Res Int (2017) 2017:2767484. 28164117 10.1155/2017/2767484 PMC5259610

[69] Sun JY Huang Y Li JP Zhang X Wang L Meng Y-L. MicroRNA-320a suppresses human colon cancer cell proliferation by directly targeting beta-catenin. Biochem Biophys Res Commun (2012) 420:787–92. doi: 10.1016/j.bbrc.2012.03.075 22459450

[70] Wu F Xing T Gao X Liu F. miR5013p promotes colorectal cancer progression via activation of wnt/betacatenin signaling. Int J Oncol (2019) 55:671–83. 10.3892/ijo.2019.4852 PMC6685591 31364752

[71] Fasihi A Soltani BM Ranjbaran ZS Ihonar S Norouzi R Nasiri S. Hsa-miR-942 fingerprint in colorectal cancer through wnt signaling pathway. Gene (2019) 712:143958. doi: 10.1016/j.gene.2019.143958 31278963

[72] Shan Z An N Qin J Yang J Sun H Yang W. Long non-coding RNA Linc00675 suppresses cell proliferation and metastasis in colorectal cancer via acting on miR-942 and wnt/beta-catenin signaling. Biomed pharmacother = Biomed pharmacotherapie (2018) 101:769–76. doi: 10.1016/j.biopha.2018.02.123 29524886

[73] Zhang Y Guo L Li Y. MicroRNA-494 promotes cancer progression and targets adenomatous polyposis coli in colorectal cancer. Mol canc (2018) 17:1. doi: 10.1186/s12943-017-0753-1 PMC5755155 29304823

[74] Geng Y Zheng X Hu W. Hsa_circ_0009361 acts as the sponge of miR-582 to suppress colorectal cancer progression by regulating APC2 expression. Clin Sci (London Engl 1979). (2019) 133:1197–213. doi: 10.1042/CS20190286 31109967

[75] Nagel R le Sage C Diosdado B. Regulation of the adenomatous polyposis coli gene by the miR-135 family in colorectal cancer. Cancer Res (2008) 68:5795–802. doi: 10.1158/0008-5472.CAN-08-0951 18632633

[76] Xiao F Chen W Yu C Zhao G. MicroRNA-663b enhances migration and invasion by targeting adenomatous polyposis coli 2 in colorectal carcinoma cells. Oncol Lett (2020) 19:3701–10. doi: 10.3892/ol.2020.11482 PMC7202279 32382323

[77] Fasihi A MS B Ishi A NasiI S. IntroducIn of hsa-miR-103a and hsa-miR-1827 and hsa-miR-137 as new regulators of wnt signaling pathway and their relation to colorectal carcinoma. J Cell Biochem (2018) 119:5104–17. doi: 10.1002/jcb.26357 28817181

[78] Chen HY Lang YD Lin HN. miR-103/107 prolong wnt/beta-catenin signaling and colorectal cancer stemness by targeting Axin2. Sci Rep (2019) 9:9687. doi: 10.1038/s41598-019-41053-z 31273221 PMC6609830

[79] Kim NH Cha YH Kang SE Lee Ym Lee I Cha SY. p53 regulates nuclear GSK-3 levels through miR-34-mediated Axin2 suppression in colorectal cancer cells. Cell Cycle (Georgetown Tex) (2013) 12:1578–87. doi: 10.4161/cc.24739 PMC3680537 23624843

[80] Zhang GJ Li LF Yang GD. MiR-92a promotes stem cell-like properties by activating wnt/beta-catenin signaling in colorectal cancer. Oncotarget (2017) 8:101760–70. doi: 10.18632/oncotarget.21667 PMC5731912 29254202

[81] Li T Lai Q Wang S. MicroRNA-224 sustains wnt/beta-catenin signaling and promotes aggressive phenotype of colorectal cancer. J Exp Clin Cancer Res CR. (2016) 35:21. doi: 10.1186/s13046-016-0287-1 26822534 PMC4731927

[82] Sun N Zhang G Liu Y. Long non-coding RNA XIST sponges miR-34a to promotes colon cancer progression via wnt/beta-catenin signaling pathway. Gene (2018) 665:141–8. doi: 10.1016/j.gene.2018.04.014 29679755

[83] Li L Wang A Cai M Tong M Chen F Huang L. Identification of stool miR-135b-5p as a non-invasive diaognostic biomarker in later tumor stage of colorectal cancer. Life Sci (2020) 260:118417. doi: 10.1016/j.lfs.2020.118417 32931801

[84] Si G Li S Zheng Q Zhu S Zhou C. miR-1246 shuttling from fibroblasts promotes colorectal cancer cell migration. Neoplasma (2021) 68:317–24. doi: 10.4149/neo_2020_200924N1018 33231089

[85] Santos EDS Nogueira KAB Fernandes LCC. EGFR targeting for cancer therapy: pharmacology and immunoconjugates with drugs and nanoparticles. Int J pharmaceutics (2021) 592:120082. doi: 10.1016/j.ijpharm.2020.120082 33188892

[86] Neumann J Zeindl-Eberhart E Kirchner T Jung A. Frequency and type of KRAS mutations in routine diagnostic analysis of metastatic colorectal cancer. Pathology Res practice (2009) 205:858–62. doi: 10.1016/j.prp.2009.07.010 19679400

[87] Chang DZ Kumar V Ma Y Li K Kopetz S. Individualized therapies in colorectal cancer: KRAS as a marker for response to EGFR-targeted therapy. J Hematol Oncol (2009) 2:18. doi: 10.1186/1756-8722-2-18 19386128 PMC2686726

[88] Zhang Q Jeppesen DK Higginbotham JN. Mutant KRAS exosomes alter the metabolic state of recipient colonic epithelial cells. Cell Mol Gastroenterol hepatology (2018) 5:627–629.e626. doi: 10.1016/j.jcmgh.2018.01.013 PMC6009797 29930982

[89] Du F Cao T Xie H. KRAS mutation-responsive miR-139-5p inhibits colorectal cancer progression and is repressed by wnt signaling. Theranostics (2020) 10:7335–50. doi: 10.7150/thno.45971 PMC7330859 32641995

[90] Saridaki Z Weidhaas JB Lenz HJ. A let-7 microRNA-binding site polymorphism in KRAS predicts improved outcome in patients with metastatic colorectal cancer treated with salvage cetuximab/panitumumab monotherapy. Clin Cancer Res an Off J Am Assoc Cancer Res (2014) 20:4499–510. doi: 10.1158/1078-0432.CCR-14-0348 PMC4155520 25183481

[91] You C Liang H Sun W. Deregulation of the miR-16-KRAS axis promotes colorectal cancer. Sci Rep (2016) 6:37459. doi: 10.1038/srep37459 27857191 PMC5114589

[92] Shen H Xing C Cui K. MicroRNA-30a attenuates mutant KRAS-driven colorectal tumorigenesis via direct suppression of ME1. Cell Death differentiation (2017) 24:1253–62. doi: 10.1038/cdd.2017.63 PMC5520171 28475173

[93] Liao WT Ye YP Zhang NJ. MicroRNA-30b functions as a tumor suppressor in human colorectal cancer by targeting KRAS, PIK3CD and BCL2. J pathology (2014) 232:415–27. doi: 10.1002/path.4309 24293274

[94] Kent OA Chivukula RR Mullendore M. Repression of the miR-143/145 cluster by oncogenic ras initiates a tumor-promoting feed-forward pathway. Genes Dev (2010) 24:2754–9. doi: 10.1101/gad.1950610 PMC3003192 21159816

[95] Wang YX Chen YR Liu SS. MiR-384 inhibits human colorectal cancer metastasis by targeting KRAS and CDC42. Oncotarget (2016) 7:84826–38. doi: 10.18632/oncotarget.12704 PMC5356701 27769041

[96] Fang Y Sun B Li Z Chen Z Xiang J. MiR-622 inhibited colorectal cancer occurrence and metastasis by suppressing K-ras. Mol carcinogenesis (2016) 55:1369–77. doi: 10.1002/mc.22380 26333174

[97] Pei Q Liu GS Li HP. Long noncoding RNA SNHG14 accelerates cell proliferation, migration, invasion and suppresses apoptosis in colorectal cancer cells by targeting miR-944/KRAS axis through PI3K/AKT pathway. Eur Rev Med Pharmacol Sci (2019) 23:9871–81. 10.26355/eurrev_201911_19551 31799655

[98] Kasprzak A. Insulin-like growth factor 1 (IGF-1) signaling in glucose metabolism in colorectal cancer. Int J Mol Sci (2021) 22. doi: 10.3390/ijms22126434 PMC8234711 34208601

[99] Toda K Kawada K Iwamoto M. Metabolic alterations caused by KRAS mutations in colorectal cancer contribute to cell adaptation to glutamine depletion by upregulation of asparagine synthetase. Neoplasia (New York NY). (2016) 18:654–65. doi: 10.1016/j.neo.2016.09.004 PMC5071549 27764698

[100] Xu H Liu Y Cheng P. CircRNA_0000392 promotes colorectal cancer progression through the miR-193a-5p/PIK3R3/AKT axis. J Exp Clin Cancer Res CR. (2020) 39:283. doi: 10.1186/s13046-020-01799-1 33317596 PMC7735421

[101] Jiang T Wang H Liu L. CircIL4R activates the PI3K/AKT signaling pathway via the miR-761/TRIM29/PHLPP1 axis and promotes proliferation and metastasis in colorectal cancer. Mol canc (2021) 20:167. doi: 10.1186/s12943-021-01474-9 PMC8684286 34922544

[102] Tang Y Weng X Liu C Li X Chen C. Hypoxia enhances activity and malignant behaviors of colorectal cancer cells through the STAT3/MicroRNA-19a/PTEN/PI3K/AKT axis. Analytical Cell Pathol (Amsterdam). (2021) 2021:4132488. doi: 10.1155/2021/4132488 PMC8595003 34796092

[103] Wang N He X Zhou R Jia G Qiao Q. STAT3 induces colorectal carcinoma progression through a novel miR-572-MOAP-1 pathway. Onco Targets Ther (2018) 11:3475–84. doi: 10.2147/OTT.S158764 PMC6007208 29942139

[104] Ma J Yang Y Fu Y. PIAS3-mediated feedback loops promote chronic colitis-associated malignant transformation. Theranostics (2018) 8:3022–37. doi: 10.7150/thno.23046 PMC5996365 29896300

[105] Neuzillet C Tijeras-Raballand A Cohen R. Targeting the TGFbeta pathway for cancer therapy. Pharmacol Ther (2015) 147:22–31. doi: 10.1016/j.pharmthera.2014.11.001 25444759

[106] Bao Y Chen Z Guo Y. Tumor suppressor microRNA-27a in colorectal carcinogenesis and progression by targeting SGPP1 and Smad2. PloS One (2014) 9:e105991. doi: 10.1371/journal.pone.0105991 25166914 PMC4148394

[107] Xu Q Tong JL Zhang CP Xiao Q Lin XL Xiao XY. miR-27a induced by colon cancer cells in HLECs promotes lymphangiogenesis by targeting SMAD4. PloS One (2017) 12:e0186718. doi: 10.1371/journal.pone.0186718 29065177 PMC5655427

[108] Zhai H Fesler A Ba Y Wu S Ju J. Inhibition of colorectal cancer im cell survival and invasive potential by hsa-miR-140-5p mediated suppression of Smad2 and autophagy. Oncotarget (2015) 6:19735–46. doi: 10.18632/oncotarget.3771 PMC4637317 25980495

[109] Sun C Wang FJ Zhang HG. miR-34a mediates oxaliplatin resistance of colorectal cancer cells by inhibiting macroautophagy via transforming growth factor-β/Smad4 pathway. World J gastroenterology (2017) 23:1816–27. doi: 10.3748/wjg.v23.i10.1816 PMC5352922 28348487

[110] Dews M Fox JL Hultine S. The myc-miR-17~92 axis blunts TGF{beta} signaling and production of multiple TGF{beta}-dependent antiangiogenic factors. Cancer Res (2010) 70:8233–46. doi: 10.1158/0008-5472.CAN-10-2412 PMC3007123 20940405

[111] Zhao X Liu S Yan B Yang J Chen E. MiR-581/SMAD7 axis contributes to colorectal cancer metastasis: a bioinformatic and experimental validation-based study. Int J Mol Sci (2020) 21. doi: 10.3390/ijms21186499 PMC7555590 32899503

[112] Bu P Wang L Chen KY. miR-1269 promotes metastasis and forms a positive feedback loop with TGF-β. Nat Commun (2015) 6:6879. doi: 10.1038/ncomms7879 25872451 PMC4399006

[113] Zhang Q Wang J Li N. miR-34a increases the sensitivity of colorectal cancer cells to 5-fluorouracil *in vitro* and *in vivo* . Am J Cancer Res (2018) 8:280–90. PMC5835695 29511598

[114] Jin Y Wang M Hu H Huang Q Chen Y Wang G. Overcoming stemness and chemoresistance in colorectal cancer through miR-195-5p-modulated inhibition of notch signaling. Int J Biol macromolecules (2018) 117:445–53. doi: 10.1016/j.ijbiomac.2018.05.151 29852230

[115] Wang XW Xi XQ Wu J Wan YY Hui HX Cao XF. MicroRNA-206 attenuates tumor proliferation and migration involving the downregulation of NOTCH3 in colorectal cancer. Oncol Rep (2015) 33:1402–10. doi: 10.3892/or.2015.3731 25607234

[116] Chen J Zhang H Chen Y. miR-598 inhibits metastasis in colorectal cancer by suppressing JAG1/Notch2 pathway stimulating EMT. Exp Cell Res (2017) 352:104–12. doi: 10.1016/j.yexcr.2017.01.022 28161537

[117] Liu T Liu D Guan S Dong M. Diagnostic role of circulating MiR-21 in colorectal cancer: a update meta-analysis. Ann Med (2021) 53:87–102. doi: 10.1080/07853890.2020.1828617 33108223 PMC7877941

[118] Chang PY Chen CC Chang YS. MicroRNA-223 and microRNA-92a in stool and plasma samples act as complementary biomarkers to increase colorectal cancer detection. Oncotarget (2016) 7:10663–75. doi: 10.18632/oncotarget.7119 PMC4891149 26848774

[119] Zhang H Zhu M Shan X. A panel of seven-miRNA signature in plasma as potential biomarker for colorectal cancer diagnosis. Gene (2019) 687:246–54. doi: 10.1016/j.gene.2018.11.055 30458288

[120] Tan Y Lin JJ Yang X. A panel of three plasma microRNAs for colorectal cancer diagnosis. Cancer Epidemiol (2019) 60:67–76. doi: 10.1016/j.canep.2019.01.015 30925282

[121] Rapado-Gonzalez O Majem B Alvarez-Castro A. A novel saliva-based miRNA signature for colorectal cancer diagnosis. J Clin Med (2019) 8. doi: 10.3390/jcm8122029 PMC6947363 31757017

[122] Yau TO Tang CM Harriss EK Dickins B Polytarchou C. Faecal microRNAs as a non-invasive tool in the diagnosis of colonic adenomas and colorectal cancer: a meta-analysis. Sci Rep (2019) 9:9491. doi: 10.1038/s41598-019-45570-9 31263200 PMC6603164

[123] Sur D Advani S Braithwaite D. MicroRNA panels as diagnostic biomarkers for colorectal cancer: a systematic review and meta-analysis. Front Med (2022) 9:915226. doi: 10.3389/fmed.2022.915226 PMC9676370 36419785

[124] Boussios S Ozturk MA Moschetta M. The developing story of predictive biomarkers in colorectal cancer. J personalized Med (2019) 9. doi: 10.3390/jpm9010012 PMC6463186 30736475

[125] Chen B Xia Z Deng YN. Emerging microRNA biomarkers for colorectal cancer diagnosis and prognosis. Open Biol (2019) 9:180212. doi: 10.1098/rsob.180212 30958116 PMC6367136

[126] Vu T Datta PK. Regulation of EMT in colorectal cancer: a culprit in metastasis. Cancers (2017) 9. doi: 10.3390/cancers9120171 PMC5742819 29258163

[127] Zhao S Sun H Jiang W. miR-4775 promotes colorectal cancer invasion and metastasis via the Smad7/TGFbeta-mediated epithelial to mesenchymal transition. Mol canc (2017) 16:12. doi: 10.1186/s12943-017-0585-z PMC5240405 28095858

[128] Wang H Yan B Zhang P. MiR-496 promotes migration and epithelial-mesenchymal transition by targeting RASSF6 in colorectal cancer. J Cell Physiol (2020) 235:1469–79. doi: 10.1002/jcp.29066 31273789

[129] Przygodzka P Papiewska-Pajak I Bogusz-Koziarska H Sochacka E Boncela J Kowalska MA. Regulation of miRNAs by snail during epithelial-to-mesenchymal transition in HT29 colon cancer cells. Sci Rep (2019) 9:2165. doi: 10.1038/s41598-019-39200-7 30770873 PMC6377707

[130] Niu L Yang W Duan L. Biological implications and clinical potential of metastasis-related miRNA in colorectal cancer. Mol Ther Nucleic Acids (2021) 23:42–54. doi: 10.1016/j.omtn.2020.10.030 33335791 PMC7723777

[131] Saberinia A Alinezhad A Jafari F Soltany S Akhavan Sigari R. Oncogenic miRNAs and target therapies in colorectal cancer. Clinica chimica acta; Int J Clin Chem (2020) 508:77–91. doi: 10.1016/j.cca.2020.05.012 32407782

[132] Zhou Q Zhu Y Wei X. MiR-590-5p inhibits colorectal cancer angiogenesis and metastasis by regulating nuclear factor 90/vascular endothelial growth factor a axis. Cell Death disease (2016) 7:e2413. doi: 10.1038/cddis.2016.306 27735951 PMC5133975

[133] Chen X Zeng K Xu M. P53-induced miR-1249 inhibits tumor growth, metastasis, and angiogenesis by targeting VEGFA and HMGA2. Cell Death disease (2019) 10:131. doi: 10.1038/s41419-018-1188-3 30755600 PMC6372610

[134] Fang Y Sun B Wang J Wang Y. miR-622 inhibits angiogenesis by suppressing the CXCR4-VEGFA axis in colorectal cancer. Gene (2019) 699:37–42. doi: 10.1016/j.gene.2019.03.004 30851425

[135] Hong S Chen S Wang X. ATAD2 silencing decreases VEGFA secretion through targeting has-miR-520a to inhibit angiogenesis in colorectal cancer. Biochem Cell Biol = Biochimie biologie cellulaire. (2018) 96:761–8. doi: 10.1139/bcb-2018-0081 29958090

[136] Xu Z Zhu C Chen C. CCL19 suppresses angiogenesis through promoting miR-206 and inhibiting Met/ERK/Elk-1/HIF-1alpha/VEGF-A pathway in colorectal cancer. Cell Death disease (2018) 9:974. doi: 10.1038/s41419-018-1010-2 30250188 PMC6155262

[137] Yamakuchi M Lotterman CD Bao C. P53-induced microRNA-107 inhibits HIF-1 and tumor angiogenesis. Proc Natl Acad Sci United States America. (2010) 107:6334–9. doi: 10.1073/pnas.0911082107 PMC2851979 20308559

[138] Manfredi S Bouvier AM Lepage C Hatem C Dancourt V Faivre J. Incidence and patterns of recurrence after resection for cure of colonic cancer in a well defined population. Br J surgery. (2006) 93:1115–22. doi: 10.1002/bjs.5349 16804870

[139] Kandimalla R Gao F Matsuyama T. Genome-wide discovery and identification of a novel miRNA signature for recurrence prediction in stage II and III colorectal cancer. Clin Cancer Res an Off J Am Assoc Cancer Res (2018) 24:3867–77. doi: 10.1158/1078-0432.CCR-17-3236 PMC6095767 29514841

[140] Fukada M Matsuhashi N Takahashi T. Postoperative changes in plasma miR21-5p as a novel biomarker for colorectal cancer recurrence: a prospective study. Cancer science (2021) 112:4270–80. doi: 10.1111/cas.15065 PMC8486189 34270831

[141] Yuan Z Baker K Redman MW. Dynamic plasma microRNAs are biomarkers for prognosis and early detection of recurrence in colorectal cancer. Br J Canc (2017) 117:1202–10. doi: 10.1038/bjc.2017.266 PMC5674097 28809863

[142] Matsumura T Sugimachi K Iinuma H. Exosomal microRNA in serum is a novel biomarker of recurrence in human colorectal cancer. Br J Canc (2015) 113:275–81. doi: 10.1038/bjc.2015.201 PMC4506387 26057451

[143] Liu X Xie T Mao X Xue L Chu X Chen L. MicroRNA-149 increases the sensitivity of colorectal cancer cells to 5-fluorouracil by targeting forkhead box transcription factor FOXM1. Cell Physiol Biochem Int J Exp Cell physiology biochemistry Pharmacol (2016) 39:617–29. doi: 10.1159/000445653 27415661

[144] Wan LY Deng J Xiang XJ. miR-320 enhances the sensitivity of human colon cancer cells to chemoradiotherapy *in vitro* by targeting FOXM1. Biochem Biophys Res Commun (2015) 457:125–32. doi: 10.1016/j.bbrc.2014.11.039 25446103

[145] Liu B Liu Y Zhao L. Upregulation of microRNA-135b and microRNA-182 promotes chemoresistance of colorectal cancer by targeting ST6GALNAC2 via PI3K/AKT pathway. Mol carcinogenesis (2017) 56:2669–80. doi: 10.1002/mc.22710 28767179

[146] Zhang Y Talmon G Wang J. MicroRNA-587 antagonizes 5-FU-induced apoptosis and confers drug resistance by regulating PPP2R1B expression in colorectal cancer. Cell Death disease (2015) 6:e1845. doi: 10.1038/cddis.2015.200 26247730 PMC4558495

[147] Liu N Li J Zhao Z. MicroRNA-302a enhances 5-fluorouracil-induced cell death in human colon cancer cells. Oncol Rep (2017) 37:631–9. doi: 10.3892/or.2016.5237 27840990

[148] Fu Q Cheng J Zhang J. miR-20b reduces 5-FU resistance by suppressing the ADAM9/EGFR signaling pathway in colon cancer. Oncol Rep (2017) 37:123–30. doi: 10.3892/or.2016.5259 27878272

[149] Zhou H Lin C Zhang Y. miR-506 enhances the sensitivity of human colorectal cancer cells to oxaliplatin by suppressing MDR1/P-gp expression. Cell proliferation (2017) 50. doi: 10.1111/cpr.12341 PMC6529089 28217977

[150] Fang L Li H Wang L. MicroRNA-17-5p promotes chemotherapeutic drug resistance and tumor metastasis of colorectal cancer by repressing PTEN expression. Oncotarget (2014) 5:2974–87. doi: 10.18632/oncotarget.1614 PMC4102784 24912422

[151] Zhang Y Liu X Zhang J. Inhibition of miR-19a partially reversed the resistance of colorectal cancer to oxaliplatin via PTEN/PI3K/AKT pathway. Aging (2020) 12:5640–50. doi: 10.18632/aging.102929 PMC7185119 32209726

[152] Zhou Y Wan G Spizzo R. miR-203 induces oxaliplatin resistance in colorectal cancer cells by negatively regulating ATM kinase. Mol Oncol (2014) 8:83–92. doi: 10.1016/j.molonc.2013.09.004 24145123 PMC4124530

[153] Bitarte N Bandres E Boni V. MicroRNA-451 is involved in the self-renewal, tumorigenicity, and chemoresistance of colorectal cancer stem cells. Stem Cells (Dayton Ohio) (2011) 29:1661–71. doi: 10.1002/stem.741 21948564

[154] Khorrami S Zavaran Hosseini A Mowla SJ Soleimani M Rakhshani N Malekzadeh R. MicroRNA-146a induces immune suppression and drug-resistant colorectal cancer cells. Tumor Biol J Int Soc Oncodevelopmental Biol Med (2017) 39:1010428317698365. doi: 10.1177/1010428317698365 28466779

[155] Chang HY Ye SP Pan SL. Overexpression of miR-194 reverses HMGA2-driven signatures in colorectal cancer. Theranostics (2017) 7:3889–900. doi: 10.7150/thno.20041 PMC5667412 29109785

[156] Ren LL Yan TT Shen CQ. The distinct role of strand-specific miR-514b-3p and miR-514b-5p in colorectal cancer metastasis. Cell Death disease (2018) 9:687. doi: 10.1038/s41419-018-0732-5 29880874 PMC5992212

[157] To KK Leung WW Ng SS. Exploiting a novel miR-519c-HuR-ABCG2 regulatory pathway to overcome chemoresistance in colorectal cancer. Exp Cell Res (2015) 338:222–31. doi: 10.1016/j.yexcr.2015.09.011 26386386

[158] Lu Y Zhao X Liu Q. lncRNA MIR100HG-derived miR-100 and miR-125b mediate cetuximab resistance via wnt/β-catenin signaling. Nat Med (2017) 23:1331–41. doi: 10.1038/nm.4424 PMC5961502 29035371

[159] Tsukamoto M Iinuma H Yagi T Matsuda K Hashiguchi Y. Circulating exosomal MicroRNA-21 as a biomarker in each tumor stage of colorectal cancer. Oncology (2017) 92:360–70. doi: 10.1159/000463387 28376502

[160] Ashizawa M Okayama H Ishigame T. miRNA-148a-3p regulates immunosuppression in DNA mismatch repair-deficient colorectal cancer by targeting PD-L1. Mol Cancer Res MCR. (2019) 17:1403–13. doi: 10.1158/1541-7786.MCR-18-0831 30872332

[161] Shadbad MA Asadzadeh Z Derakhshani A. A scoping review on the potentiality of PD-L1-inhibiting microRNAs in treating colorectal cancer: toward single-cell sequencing-guided biocompatible-based delivery. Biomed pharmacother = Biomed pharmacotherapie (2021) 143:112213. doi: 10.1016/j.biopha.2021.112213 34560556

[162] Yang Y Meng WJ Wang ZQ. MicroRNAs (miRNAs): novel potential therapeutic targets in colorectal cancer. Front Oncol (2022) 12:1054846. doi: 10.3389/fonc.2022.1054846 36591525 PMC9794577

[163] Wu N Fesler A Liu H Ju J. Development of novel miR-129 mimics with enhanced efficacy to eliminate chemoresistant colon cancer stem cells. Oncotarget (2018) 9:8887–97. doi: 10.18632/oncotarget.22322 PMC5823633 29507661

